# Nonsense‐Mediated Decay mRNA Quality Control System Is Essential for Root Development and Efficient Root Nodule Symbiosis in *Medicago truncatula*

**DOI:** 10.1111/pce.70683

**Published:** 2026-06-21

**Authors:** János B. Biró, Csaba Péter, Ágota Domonkos, Zoltán Hegedűs, Lóránt Lakatos, Deepali Rana, Péter Gyula, Dániel Silhavy, Péter Kaló

**Affiliations:** ^1^ HUN‐REN Biological Research Centre Institute of Plant Biology Szeged Hungary; ^2^ Institute of Genetics and Biotechnology Hungarian University of Agriculture and Life Sciences Godollo Hungary; ^3^ Bioinformatics Laboratory, Core Facility HUN‐REN Biological Research Centre Szeged Hungary; ^4^ Department of Biochemistry and Medical Chemistry, Medical School University of Pecs Pecs Hungary

## Abstract

- We investigated the role of nonsense‐mediated mRNA decay (NMD) in root nodule symbiosis using NMD‐deficient *Medicago truncatula* roots. We show that NMD is essential for root growth and efficient nodulation, likely because it regulates multiple symbiosis‐related pathways in the roots.

Although root‐nodule‐symbiosis (RNS) between legumes and nitrogen‐fixing soil bacteria (rhizobia) has been studied for decades, the role of quality control systems, which ensure the fidelity of gene expression by identifying and eliminating aberrant gene products, remains unexplored. Here, we studied the function of the nonsense‐mediated decay (NMD) mRNA quality control pathway in RNS. NMD recognizes and degrades faulty mRNAs containing premature termination codons (PTCs), as well as normal transcripts that exhibit features of aberrant mRNAs.

Under nitrogen‐limited conditions, legume roots secrete flavonoids that induce the production of nodulation factors by rhizobia. These signals initiate developmental reprogramming in the host root, enabling symbiosis. Root hairs entrap rhizobia, followed by the formation of infection threads (ITs). When ITs reach nodule primordia in the root cortex, rhizobia are released into plant cells and differentiate into nitrogen‐fixing bacteroids. RNS requires efficient signaling between partners and rapid suppression of host defense (Grundy et al. 2023; Yang et al. 2022).

NMD is a conserved translation‐termination‐coupled surveillance system that degrades mRNAs whose 3′untranslated regions (3′UTR) contain translation‐termination inhibitory elements (NMD‐features) (Carrard and Lejeune 2023). When the ribosome reaches a stop codon, the ribosome recruits the eRF1‐eRF3 (eukaryotic Release Factor 1 and 3) complex and initiates translation termination, which is enhanced by the interaction between eRF3 and PABP (Poly(A)‐Binding Protein). However, if the 3′UTR is unusually long and/or contains introns > 50nt from the stop codon (plant NMD‐features), termination will be slow, and the UPF1 (Up‐Frameshift suppressor 1), NMD factor, in place of PABP, will be recruited to the termination complex. Subsequently, UPF2 and UPF3 bind to UPF1 and facilitate its phosphorylation. Finally, SMG7 (Suppressor with Morphogenetic effect on Genitalia 7) binds to phospho‐UPF1 and induces mRNA decay. NMD targets both PTC‐containing and normal mRNAs whose 3'UTR harbors NMD‐features. In plants, two major types of NMD are distinguished based on the inducer: long 3′UTR‐ and intron‐based NMD (Figure S1). The UPF1‐2‐3 and SMG7 are required for both types of NMD, while components of the exon junction complex (EJC) function specifically in intron‐based NMD. Upstream open reading frames (uORF) in the 5′UTR could also induce NMD when they encode proteins longer than > 30 aa (Rana et al. 2026; Raxwal and Riha 2023). Plant NMD activity is controlled by multiple autoregulatory circuits. The mRNAs of SMG7 and eRF1‐1 contain NMD‐features and are subject to NMD degradation in all angiosperms (Nyikó et al. 2017). Other NMD components are also downregulated by NMD (Vexler et al. 2016).

NMD is essential in plants; *Arabidopsis upf1* and *smg7* null‐mutants are lethal. NMD in *Arabidopsis* also controls defense responses by constitutively eliminating the mRNAs of resistance (R) genes (Gloggnitzer et al. 2014). Upon *Pseudomonas* infection, AtUPF1 is degraded, leading to the accumulation of NMD‐targeted R‐gene transcripts and enhanced antibacterial defense (Jung et al. 2020). Given that suppression of host defense is essential for RNS and NMD controls antibacterial defense in *Arabidopsis*, we investigated whether NMD also plays a role in symbiosis.

We identified homologs of all core NMD and EJC factors in *M. truncatula*, suggesting that both long 3′UTR‐ and intron‐based NMD are functional in *M. truncatula*. *MtUPF3* and *MtSMG7* are present as duplicated genes, while *MtUPF1*, *MtUPF2*, and the EJC factors are single‐copy genes (Table S1). To investigate the function of NMD in symbiosis, we attempted to generate *MtUPF1* and *MtUPF2* knockout roots using CRISPR‐Cas9 gene editing system and assess their symbiotic competence. However, we failed to obtain *MtUPF1* or *MtUPF2* knockouts (Figure S2), suggesting that NMD is essential for root development (for discussion, see Supporting Information S2: Supporting materials, Supporting discussion). We therefore used *Agrobacterium rhizogenes*‐mediated RNAi to silence *MtUPF1* and *MtUPF2* in *M. truncatula* roots. NMD inactivation was effective; in both *upf1* and *upf2* RNAi roots, target mRNA levels were selectively reduced, while the *eRF1‐1* and *SMG7* NMD reporter mRNAs (Nyikó et al. 2017) were upregulated (Figure 1a–c). Their transcript accumulation was higher in *upf1* roots, suggesting that NMD‐impairment was more effective in *upf1* than *upf2* roots. Relevantly, despite NMD‐deficiency, *upf1* and *upf2* roots grew normally.

**Figure 1:**
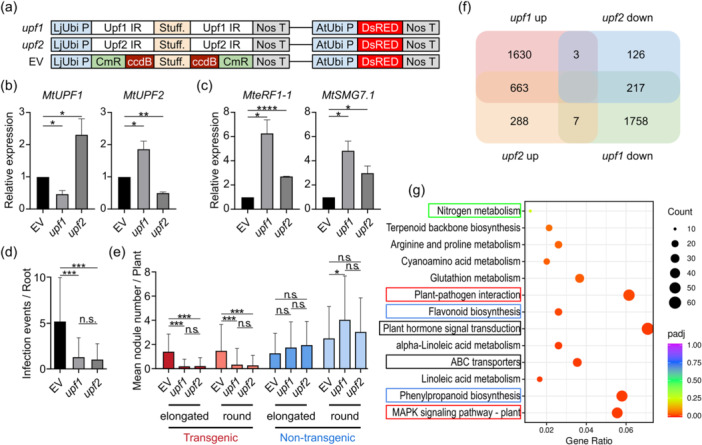
NMD‐deficiency inhibits root‐nodule‐symbiosis. (a–c) mRNA level is efficiently impaired in *upf1* and *upf2* RNAi roots. (a) RNAi constructs: inverted repeats separated by stuffer sequence (Stuff.) were cloned into a dsRED expressing vector. EV was used as negative control. (b) *MtUPF1*, *MtUPF2* target mRNAs are reduced and (c) *MtSMG7.1* and *MteRF1‐1* NMD reporter mRNAs are upregulated in *upf1* and *upf2* roots relative to the EV. (d) Early infection (4 days post inoculation) and (e) nodulation (4 weeks post inoculation) are inhibited in NMD‐deficient roots. Error bars indicate standard deviation. *, **, *** and **** indicate significance at *p* < 0.05, < 0.01, < 0.001 and < 0.0001, respectively. (f) Differentially expressed genes (DEGs) in NMD‐deficient roots. (g) NMD regulates multiple RNS‐critical KEGG pathways. Green, blue, black, and red rectangles indicate nitrogen metabolism, host plant‐rhizobia signaling, signal transduction and defense suppression related KEGG pathways enriched in *upf1* DEGs.

To assess the effect of NMD‐deficiency on RNS, *upf1*, *upf2* and control‐transformed (EV) roots were inoculated with *S. medicae*, and early infection events and nodulation were quantified. Notably, both infection events and nodule numbers were significantly reduced in NMD‐deficient roots (Figure 1d,e), indicating that NMD is required for both the early and late stages of RNS. Although NMD impairment was weaker in *upf2*, infection and nodulation were similarly reduced in both mutants. Thus, NMD efficiency is critical for RNS; even partial impairment of NMD severely compromises symbiotic competence.

To clarify how NMD‐deficiency affects symbiosis, RNA‐seq was conducted on non‐infected *upf1*, *upf2* and EV roots. NMD was found to regulate ~5%–15% of genes in *M. truncatula* roots; with 4278 and 1304 differentially expressed genes (DEGs) identified in *upf1* and *upf2* roots, respectively (Figure 1f and Table S2). There was a substantial overlap between the data set, with 70% of *upf2* DEGs were also detected in *upf1*. The higher number of DEGs in *upf1* likely reflects stronger NMD‐impairment, although additional NMD‐independent roles of UPF1 cannot be excluded.

Transcripts containing NMD‐features such as long 3′ UTR, NMD‐relevant introns and uORFs were enriched among upregulated DEGs in both *upf1* and *upf2*, indicating effective NMD inactivation and suggesting that a substantial fraction of the upregulated mRNAs represent direct NMD targets (Supporting Information S1: Figure S3 and Table S3). The ratio of Down‐/Up‐DEG, used as a proxy for assessing the secondary effect of NMD‐deficiency, was markedly higher in *upf1* (0.86 vs. 0.36), indicating that more efficient NMD inactivation led to stronger secondary effects.

To understand why RNS is impaired in NMD‐deficient roots, we analyzed enriched KEGG and GO pathways among the *upf1* and *upf2* DEGs. Notably, many KEGG pathways directly linked to RNS‐essential biological processes, such as (i) plant‐rhizobia signaling (flavonoid and phenylpropanoid biosynthesis), (ii) signal transduction (plant hormone signal transduction and ABC transporter pathways), and (iii) regulation of defense responses (plant‐pathogen interaction and MAPK signaling), were significantly altered in both mutants (Figure 1g and Supporting Information S1: Figure S4). Importantly, resistance‐gene transcripts were not up‐regulated in the mutant roots (Table S4). Next, we compared the overrepresented GO categories (Supporting Information S1: Figures S5–S7). Flavonoid biosynthesis and endopeptidase activity were enriched among the Up‐DEGs in both NMD‐deficient plants. Finally, analysis of the shared *upf1* and *upf2* DEG set revealed enrichment of GO terms related to nitrogen metabolism among the Down‐DEGs. These results demonstrate that multiple RNS‐critical pathways are regulated by NMD in *M. truncatula* roots and help explain the RNS deficiency of NMD‐impaired roots (discussed in detail in the Supporting discussion section of Supporting Information S2: Supporting Material and Supporting Information S1: Figures S8–S12). The observation that many RNS‐related pathways are similarly altered in both mutants also explains why RNS is comparably inhibited in both mutants, despite less efficient NMD impairment in *upf2*.

In *Arabidopsis*, *Pseudomonas* infection inactivates NMD, thereby causing the upregulation of NMD target transcripts (Jung et al. 2020). As rhizobia elicit a transient defense response in *M. truncatula* roots, we asked whether they also trigger NMD inactivation. However, analysis of available RNA‐seq (Poehlman et al. 2019) revealed that NMD‐sensitive mRNAs were not upregulated in roots during the early phase of symbiosis (Supporting Information S1: Figures S13–S14). These data suggest that, while NMD is required for the symbiotic competence of *M. truncatula* roots, the efficiency of NMD remains unchanged during RNS.

Although NMD‐impairment was weaker in *upf2*, RNS was similarly inhibited in *upf1* and *upf2*. As even moderate NMD‐impairment led to strong RNS inhibition, we hypothesized that NMD activity is tightly autoregulated in *M. truncatula* roots. Indeed, RT‐qPCR and RNA‐seq analysis showed (Figure 1b,c and Table S2) that the mRNA levels of many NMD factors (*MtUPF1‐2‐3*, *MtSMG7* and *MtBarentsz* EJC components) were negatively regulated by NMD. As these transcripts contain NMD‐inducing features (Table S1), they are likely direct NMD targets. Consistently, agroinfiltration‐based NMD assays confirmed that the 3'UTRs of *MtUPF1*, *MtUPF2*, *MtSMG7.1*, and *MtSMG7.2* transcripts trigger NMD (Supporting Information S1: Figures S15–S16). These results indicate that NMD activity is tightly autoregulated in *M. truncatula*.

Together, our data show that NMD is essential for root growth and RNS in *M. truncatula*. As NMD regulates multiple RNS‐critical pathways, its normal activity is required for the development of symbiotic competence in roots and remains constitutively active during RNS. Our data further suggest that various autoregulatory loops stabilize NMD in *M. truncatula*. Further studies are needed to clarify whether NMD plays a direct role in the regulation of symbiosis and to elucidate the function of other protein and mRNA quality control systems in RNS.

## Conflicts of Interest

The authors declare no conflicts of interest.

## Supporting information


**Figure S1:** Plant NMD. (a) NMD‐features in plants. Plant NMD targets and degrades mRNAs containing characteristic features, including long 3'UTR, an NMD‐relevant intron (located in the 3'UTR more than 50 nt downstream of the stop codon) or an NMD‐relevant upstream ORF (a translatable uORF encoding a protein longer than 30 amino acids). (b) Mechanism of long 3'UTR‐based NMD. (c) Mechanism of intron‐based plant NMD. Splicing leads to the deposition of EJC on the mRNA 20‐25 nt upstream from the exon‐exon junctions. The EJC is a binding platform for NMD factors UPF3 and UPF2. During translation, ribosomes remove EJCs located within the coding region (as in wild‐type mRNAs), while 3'UTR‐located EJCs promote NMD by increasing the local concentration of UPF2 and UPF3, thereby facilitating NMD complex assembly. R: ribosome, EJC: exon‐junction complex, RF1: releasing factor 1, RF3: releasing factor 3, PABP: poly(A)‐binding protein, PTC: premature termination codon. **Figure S2:** The allele frequencies of CRISPR‐edited *UPF1* and *UPF2* hairy roots. *Agrobacterium rhizogenes*‐mediated hairy root transformation was used to edit the *MtUPF1* and *MtUPF2* NMD factor genes. Transgenic roots were selected, and the frequencies of edited and non‐edited alleles were determined by sequencing. A total of 15 *upf1* and 27 *upf2* transgenic roots were sequenced, and the alleles were categorized as wild type, in‐frame edited, or frameshift‐edited alleles. Frameshift‐edited alleles are likely non‐functional, whereas in‐frame edited alleles contain short deletions that do not alter the reading frame and therefore may be either functional or non‐functional. Non‐edited wild‐type alleles are considered functional. Knockout plants would therefore contain only frameshift‐edited alleles. Notably, the highest frequencies of frameshift‐edited alleles were 67% and 68% for *UPF1* and *UPF2*, respectively, indicating that neither gene could be completely knocked out (see also Supplementary Materials and Methods and Supplementary Discussion). In addition, plants containing only in‐frame edited alleles, but no wild‐type alleles, were identified among the *upf1* mutants but not among the *upf2* mutants, suggesting that the in‐frame alleles are functional in *upf1* plants but likely non‐functional in *upf2* plants. **Figure S3:** Up‐regulated *upf1* differentially expressed genes (DEGs) possess longer 3'UTRs. The length of 3'UTRs were compared among up‐ and downregulated differentially expressed genes (Up and Down DEGs), and the non‐upregulated (Non‐Up) and non‐downregulated (Non‐Down) transcripts. Up and Down DEGs were defined as transcripts showing significant and at least twofold expression differences in the *upf1* mutant relative to the control. Non‐Up mRNAs include Down DEGs and transcripts that do not show differential expression, whereas Non‐Down mRNAs include Up DEGs and transcripts that do not show differential expression in *upf1* roots. Note that 3'UTRs of the Up DEGs (marked in red) are significantly longer than those of Down DEGs and Non‐Up or Non‐Down mRNAs, indicating that NMD represses the expression of long 3'UTR containing mRNAs. *** indicates significance at *p*<0.001. **Figure S4:** KEGG pathway enrichment analyses of differentially expressed genes of *upf1* and *upf2* RNAi roots. The top 20‐20 enriched pathways for each line are shown. Enriched and strongly enriched pathways (defined as p<0.05 and padj<0.05) are separated with a horizontal red line. Pathways that are strongly enriched in both RNAi roots are marked as **, which are strongly enriched in one plant but only enriched in the other silenced plant are marked as *, and pathways, which are only enriched in both plants are shown as +. Pathways directly associated with symbiotic interactions are highlighted with rectangles. Green, yellow, black and red rectangles denote pathways related to nitrogen status, plant signal production, signal transduction and defense suppression, respectively. **Figure S5:** GO enrichment analysis of the downregulated differentially expressed genes (Down‐DEGs) in *upf1* and *upf2* silenced roots. Comparison of top enriched Gene Ontology (GO) categories among Down‐DEGs. GO categories enriched in both *upf1* and *upf2* silenced roots are highlighted with red rectangles. Fold indicates fold enrichment. **Figure S6:** GO enrichment analysis of the up‐regulated differentially expressed genes (Up‐DEGs) in *upf1* and *upf2* silenced roots. Comparison of top enriched Gene Ontology (GO) categories among Up‐DEGs. GO categories enriched in both *upf1* and *upf2* silenced roots are highlighted with rectangles. Yellow and red rectangles indicate flavonoid biosynthesis‐related and endopeptidase activity‐related GOs, respectively. Fold indicates fold enrichment. Note that 8 out of the15 GO categories enriched in *upf1* are also enriched in *upf2*. **Figure S7:** GO enrichment analysis of the up‐ and down‐regulated differentially expressed genes (Up and Down DEGs) of the NMD core gene set. Comparison of top enriched Gene Ontology (GO) categories (fold change >7). Yellow, red and green rectangles indicate GOs related to flavonoid biosynthesis, defense and nitrogen metabolism, respectively. **Figure S8:** Analysis of the MAPK signaling KEGG pathway of *upf1* DEGs. Green boxes indicate pathway components that can be identified in *Medicago truncatula*. A given box may represent a single gene, if the factor is present as a single copy in *M. truncatula*, or multiple genes, if it is encoded by more than one gene copy. Black‐framed boxes show components whose corresponding genes were not differentially expressed in *upf1*. Red framed boxes indicate components for which the corresponding gene is an upregulated DEG (Up‐DEG) in *upf1* or in the case of multicopy genes, at least one copy is an Up‐DEG, while the others show no significant change. Green‐framed boxes indicate that at least one gene copy is downregulated (Down‐DEG), and yellow‐framed boxes indicate that both Up‐ and Down‐DEGs are present. Red, green and yellow labeled characters for *upf2* indicate if the corresponding factor is an Up‐DEG, a Down‐DEG, or shows mixed regulation in *upf2*. Notably, although fewer components are altered in *upf2*, most affected components show similar expression changes in both *upf1* and *upf2* RNAi roots. **Figure S9:** Analyzes of Nitrogen Metabolism KEGG pathway of *upf1* DEGs. Symbols are described at Figure S7. **Figure S10:** Analyzes of Flavonoid biosynthesis KEGG pathway of *upf1* DEGs. Symbols are described at Figure S7. **Figure S11:** Analyzes of Linoleic Acid metabolism KEGG pathway of *upf1* DEGs. Symbols are described at Figure S7. **Figure S12:** Analyzes of Alfa Linoleic Acid metabolism KEGG pathway of *upf1* DEGs. Symbols are described at Figure S7. **Figure S13:** Expression of NMD factor genes during the early phase of nodulation. Raw sequencing data were retrieved from the SRA database (NCBI Bioproject ID: PRJNA524899) and analyzed as described in the Materials and Methods section. Roots of *M. truncatula* plants were inoculated either with mock (Control) or *S. medicae* bacteria (Inoculated) and samples were collected at the indicated time points in three biological replicates. Relative expression values are in Transcript per Million (TPM) units. Error bars represent standard deviation. The results of two‐way ANOVA tests are shown in the table. **Figure S14:** Expression of *upf1* DEGs during the early phase of root nodule symbiosis (RNS). (a) The dataset used is the same as in Figure S13. In the upper panel, the expression profiles of genes differentially expressed (DEGs) in *upf1* RNAi roots are shown. Upregulated and downregulated genes (Up‐DEG and Down‐DEG, respectively), along with 2,000 randomly selected genes are displayed as heatmaps (genes not expressed in this dataset were excluded). Colors represent Z‐scores. In the lower panel, the mean Z‐score of the genes shown in the heatmaps were calculated and plotted to assess overall expression trends within each gene set. Shaded areas represent standard deviation. Contrary to the hypothesis that NMD is inhibited during nodulation, an effect that would be expected to lead to accumulation of NMD targets, the *upf1* Up‐DEGs exhibited a slight downward trend in inoculated roots at 24h post‐inoculation compared with mock‐treated controls. The randomly selected genes did not display any consistent pattern. (b) As is panel (a) but restricted to genes whose expression is significantly affected by the interaction between Time and Treatment. To identify genes specifically responding to inoculation over time, a Likelihood Ratio Test was applied. Only a small subset of *upf1* DEGs (32 Up and 21 Down) exhibited significant expression changes. The more pronounced opposing trends observed in these filtered sets further support the conclusion that NMD remains active, or even enhanced, during the early stages of nodulation. **Figure S15:** The 3'UTRs of *MtUPF1* and *MtUPF2* induce NMD. (a) Schematic, non‐proportional representation of reporter constructs. The terminator regions of *MtUPF1* and *MtUPF2* were cloned downstream of a GFP reporter (G‐U1T and G‐U2T). (b) These constructs were agroinfiltrated into *Nicotiana benthamiana* leaves in the presence (U1DN) or absence (‐) of the dominant‐negative version of *Arabidopsis* UPF1. The P14 silencing suppressor was co‐infiltrated and used as normalization control. GFP600, a previously described NMD sensitive reporter, was used as positive control. Photographs were taken and RNA samples were collected at 3 days post‐infiltration. RNA gel blots were hybridized with P14 and GFP probes. (c) For quantification, the reporter mRNA signal (GFP probe) in each lane was normalized to the corresponding P14 signal (GFP/P14 signal). Mean values were calculated from three independent replicates. The GFP/P14 ratio of the U1DN co‐infiltrated sample was set to 1, and the GFP/P14 ratio of the (‐) sample is shown relative to this value. Error bars represent standard deviation. Student's t‐test was used to assess significance. **Figure S16:** The 3'UTR of *MtSMG7.1* and *MtSMG7.2* activate NMD. (a) The terminator regions of *MtSMG7.1* and *MtSMG7.2* were cloned downstream of a GFP reporter (G‐S7.1T and G‐S7.2T). Introns (I.) located in the 3'UTR are indicated with black I. and red I. letters represent non‐NMD relevant (located < 50 nt from the stop codon) and NMD‐relevant introns (> 50 nt from the stop codon) introns, respectively. (b) The reporter constructs were agroinfiltrated into *Nicotiana benthamiana* leaves in the presence (U1DN) or absence (‐) of the dominant‐negative version of *Arabidopsis* UPF1. GFP600, a previously described NMD sensitive reporter, was used as a positive control. The P14 silencing suppressor was co‐infiltrated and used as normalization control. Note that both 3'UTR were sensitive to NMD although the *SMG7.1* terminator showed relatively low efficiency in *N. benthamiana* leaves*.*

**Table S1:** The putative *M. truncatula* NMD factors.

**Table S2:** List of DEGs.

**Table S3:** NMD‐features of the *upf1* and *upf2* DEGs.

**Table S4:** Resistance (R‐) genes are not overrepresented in the *upf1* Up‐DEGs.

Supporting File

## Data Availability

The data that support the findings of this study are openly available in GEO repository at https://www.ncbi.nlm.nih.gov/geo/query/acc.cgi?acc=GSE320113.reference.number.GSE320113.
