# Supplementary material for: Nonsense‐Mediated Decay mRNA Quality Control System Is Essential for Root Development and Efficient Root Nodule Symbiosis in Medicago truncatula
Source: Plant Cell Environ. 2026 Jun 21;49(9):7210–4. doi: 10.1111/pce.70683 (PMC13436451; doi:10.1111/pce.70683)
Supplement: Supplementary file 1 [file PCE-49-7210-s001.pdf]

Plant NMD I.

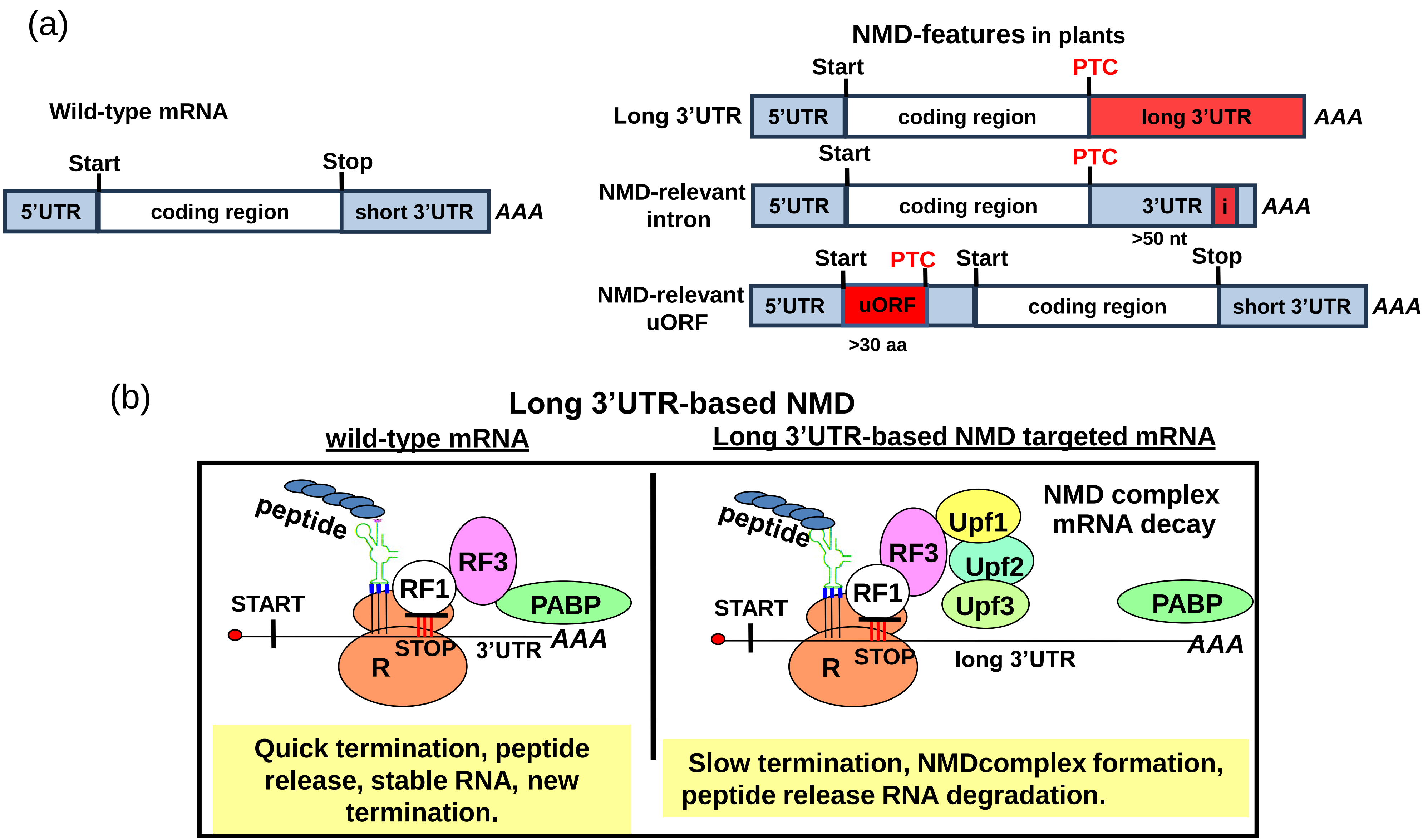

Figure S1 Plant NMD. (a) NMD-features in plants. Plant NMD targets and degrades mRNAs containing characteristic features, including long 3'UTR, an NMD-relevant intron (located in the 3'UTR more than 50 nt downstream of the stop codon) or an NMD-relevant upstream ORF (a translatable uORF encoding a protein longer than 30 amino acids). (b) Mechanism of long 3'UTR-based NMD.

Plant NMD II.

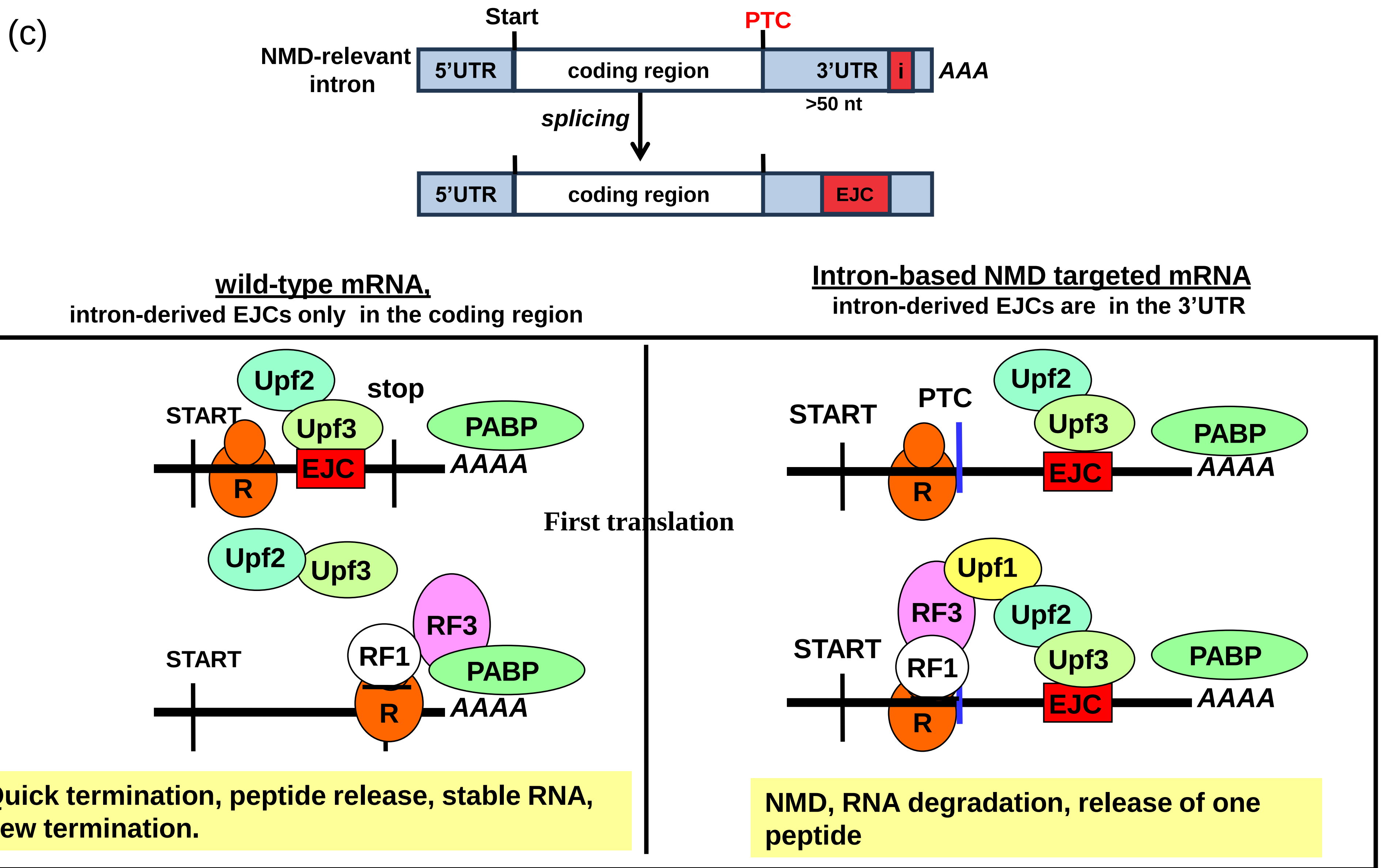

Figure S1 Plant NMD**.** (c) Mechanism of intron-based plant NMD. Splicing leads to the deposition of EJC on the mRNA 20-25 nt upstream from the exon-exon junctions. The EJC is a binding platform for NMD factors UPF3 and UPF2. During translation, ribosomes remove EJCs located within the coding region (as in wild-type mRNAs), while 3'UTR-located EJCs promote NMD by increasing the local concentration of UPF2 and 3, thereby facilitating NMD complex assembly.
R: ribosome, EJC: exon-junction complex, RF1: releasing factor 1, RF3: releasing factor 3, PABP: poly(A)-binding protein, PTC: premature termination codon

upf1

Frameshift edited allele frequency	In frame edited allele frequency	Non-edited, wild type allele frequency
67%	0%	33%
64%	36%	0%
62%	15%	23%
59%	1%	40%
58%	39%	3%
53%	47%	0%
51%	49%	0%
48%	52%	0%
48%	52%	0%
46%	54%	0%
46%	52%	2%
46%	43%	11%
39%	31%	30%
1%	67%	32%
0%	0%	100%

upf2

Frameshift edited allele frequency	In frame edited allele frequency	Non-edited, wild type allele frequency
68%	9%	23%
56%	10%	33%
53%	10%	37%
54%	7%	38%
49%	10%	41%
46%	9%	45%
46%	8%	46%
46%	8%	46%
44%	9%	47%
43%	7%	49%
41%	9%	49%
40%	9%	51%
43%	5%	52%
41%	6%	53%
35%	6%	59%
34%	5%	62%
32%	5%	62%
31%	5%	64%
35%	0%	65%
29%	5%	66%
30%	4%	66%
27%	5%	68%
25%	5%	70%
25%	4%	70%
26%	3%	72%
18%	0%	82%
13%	0%	87%

Figure S2 The allele frequencies of CRISPR edited *UPF1* and *UPF2* hairy roots. *Agrobacterium rhizogenes*-mediated hairy root transformation was used to edit the *MtUPF1* and *MtUPF2* NMD factor genes. Transgenic roots were selected, and the frequencies of edited and non-edited alleles were determined by sequencing. A total of 15 *upf1* and 27 *upf2* transgenic roots were sequenced, and the alleles were categorized as wild type, in-frame edited, or frameshift-edited alleles. Frameshift-edited alleles are likely non-functional, whereas in-frame edited alleles contain short deletions that do not alter the reading frame and therefore may be either functional or non-functional. Non-edited wild-type alleles are considered functional. Knockout plants would therefore contain only frameshift-edited alleles. Notably, the highest frequencies of frameshift-edited alleles were 67% and 68% for *UPF1* and *UPF2*, respectively, indicating that neither gene could be completely knocked out (see also Supplementary Materials and Methods and Supplementary Discussion). In addition, plants containing only in-frame edited alleles, but no wild-type alleles, were identified among the *upf1* mutants but not among the *upf2* mutants, suggesting that the in-frame alleles are functional in *upf1* plants but likely non-functional in *upf2* plants.

3'UTR statistics of *upf1* DEGs

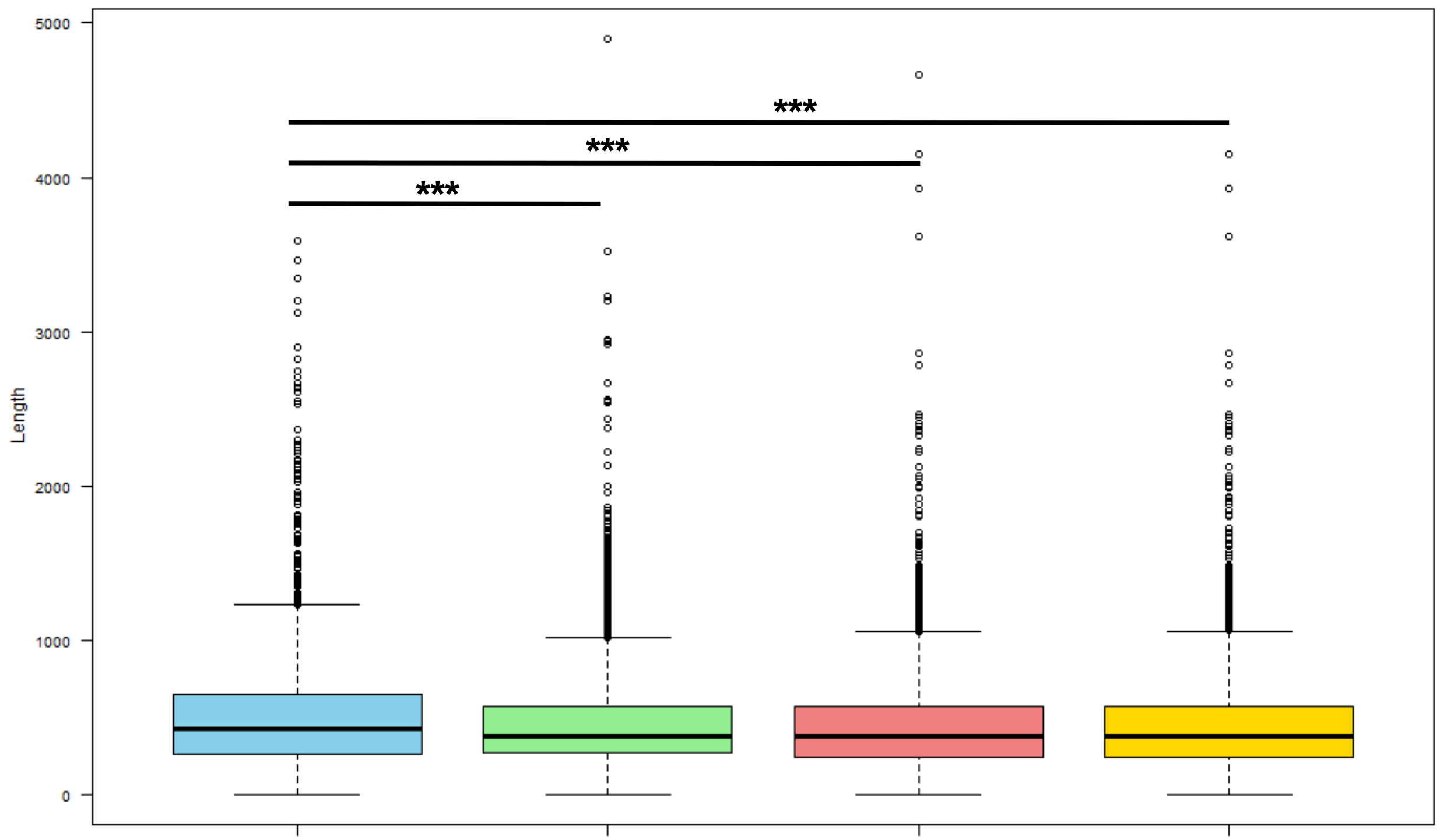

	Up DEGs	Down DEGs	Non-Up mRNAs	Non-Down mRNAs
**Mean 3'UTR length**	**532,13**	**482,09**	**475,51**	**480,29**
**Median 3'UTR length**	**423**	**375,5**	**378**	**382**
**Standard Deviation**	**440,25**	**378,16**	**408,36**	**414,20**
**Number of transcripts**	**2184**	**1980**	**22936**	**23188**

Figure S3 Up-regulated *upf1* differentially expressed genes (DEGs) possess longer 3'UTRs. The length of 3'UTRs were compared among up- and downregulated differentially expressed genes (Up and Down DEGs), and the non-upregulated (Non-Up) and non-downregulated (Non-Down) transcripts. Up and Down DEGs were defined as transcripts showing significant and at least twofold expression differencies in the *upf1* mutant relative to the control. Non-Up mRNAs include Down DEGs and transcripts that do not show differential expression, whereas Non-Down mRNAs include Up DEGs and transcripts that do not show differential expression in *upf1* roots. Note that 3'UTRs of the Up DEGs (marked in red) are significantly longer than those of Down DEGs and Non-Up or Non-Down mRNAs, indicating that NMD represses the expression of long 3'UTR containing mRNAs. *** indicates significance at $p<0.001$.

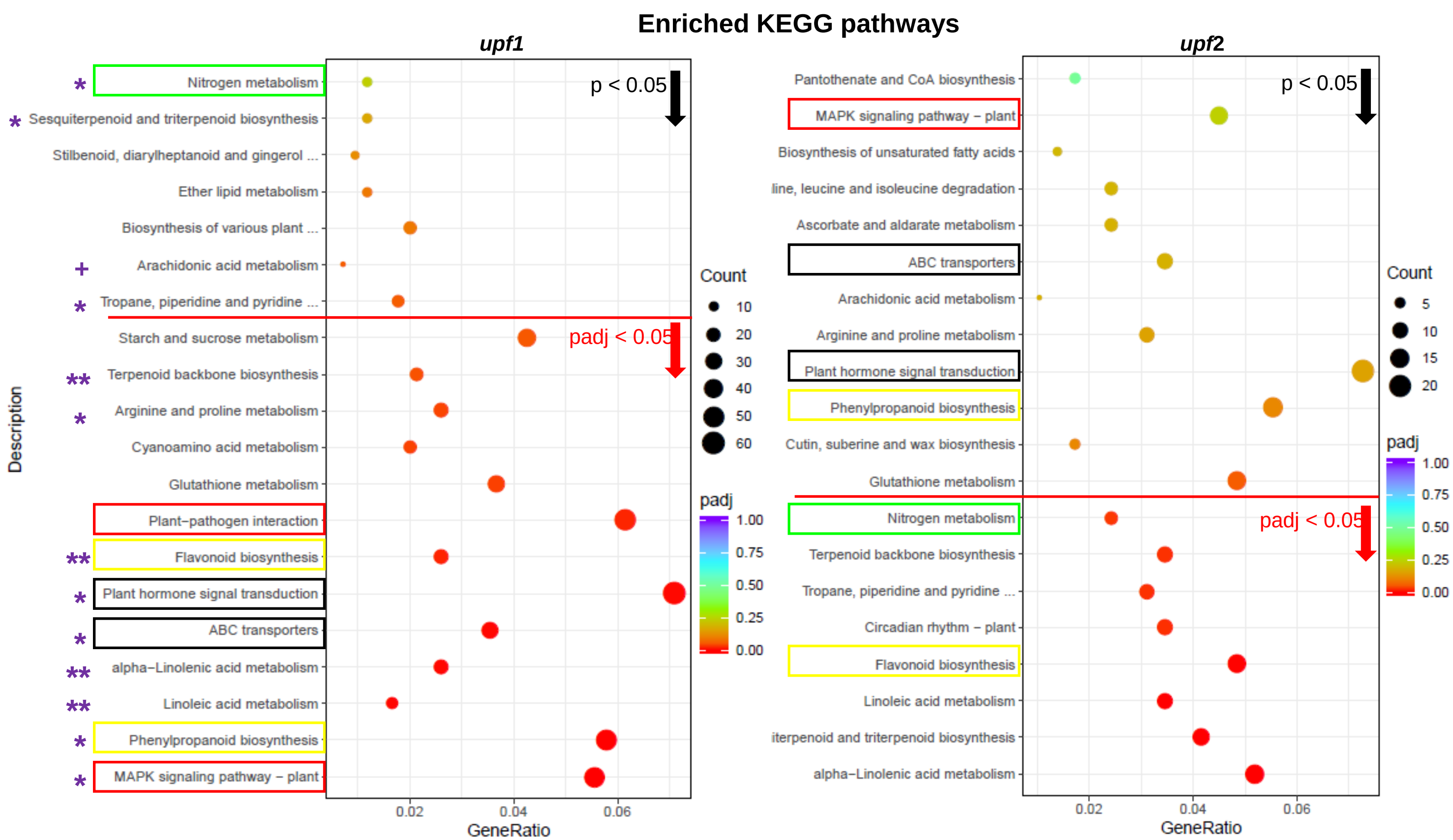

Figure S4 KEGG pathway enrichment analyses of differentially expressed genes of *upf1* and *upf2* RNAi roots. The top 20-20 enriched pathways for each line are shown. Enriched and strongly enriched pathways (defined as p<0.05 and padj<0.05) are separated with a horizontal red line. Pathways that are strongly enriched in both RNAi roots are marked as **, which are strongly enriched in one plant but only enriched in the other silenced plant are marked as *, and pathways, which are only enriched in both plants are shown as +. Pathways directly associated with symbiotic interactions are highlighted with rectangles. Green, yellow, black and red rectangles denote pathways related to nitrogen status, plant signal production, signal transduction and defense suppression, respectively.

upf1

Fold	GO category
9.6	GO:0042546 Cell wall biogenesis
9.4	GO:0016762 Xyloglucan:xyloglucosyl transferase activity
8.7	GO:0010411 Xyloglucan metabolic process
6.9	GO:0004553 Hydrolase activity-hydrolyzing O-glycosyl compounds
5.2	GO:0042744 Hydrogen peroxide catabolic process
4.8	GO:0009505 Plant-type cell wall
4.8	GO:0004601 Peroxidase activity
4.6	GO:0006979 Response to oxidative stress
3.9	GO:0098869 Cellular oxidant detoxification
3.7	GO:0046658 Anchored component of plasma membrane
3.4	GO:0005618 Cell wall
3.1	GO:0007165 Signal transduction
2.9	GO:0006952 Defense response
2.9	GO:0043565 Sequence-specific DNA binding
2.8	GO:0003700 Transcription factor activity-sequence-specific DNA binding
2.4	GO:0005576 Extracellular region
2.1	GO:0006355 Reg. of transcription-DNA-templated

upf2

Fold	GO category
101.9	GO:0006809 Nitric oxide biosynthetic process
101.9	GO:0050464 Nitrate reductase (NADPH) activity
91.7	GO:0045174 Glutathione dehydrogenase (ascorbate) activity
87.3	GO:0004737 Pyruvate decarboxylase activity
65.5	GO:0051903 S-(hydroxymethyl)glutathione dehydrogenase activity
57.3	GO:0009263 Deoxyribonucleotide biosynthetic process
43.7	GO:0030976 Thiamine pyrophosphate binding
41.7	GO:0004748 Ribonucleoside-diphosphate reductase activity-thioredoxin dis
32.7	GO:0004024 Alcohol dehydrogenase activity-zinc-dependent
27	GO:0046294 Formaldehyde catabolic process
26.6	GO:0005200 Structural constituent of cytoskeleton
14.9	GO:0000226 Microtubule cytoskeleton organization
14.9	GO:0030570 Pectate lyase activity
9.2	GO:0006334 Nucleosome assembly
7.5	GO:0045490 Pectin catabolic process
5.5	GO:0005618 Cell wall
5.5	GO:0098869 Cellular oxidant detoxification
3.1	GO:0005576 Extracellular region
2.7	GO:0003700 Transcription factor activity-sequence-specific DNA binding

Figure S5 GO enrichment analysis of the downregulated differentially expressed genes (Down-DEGs) in *upf1*and *upf2* silenced roots. Comparison of top enriched Gene Ontology (GO) categories among Down-DEGs. GO categories enriched in both *upf1*and *upf2* silenced roots are highlighted with red rectangles. Fold indicates fold enrichment.

upf1

Fold	GO category
18.5	GO:0047987 Hydroperoxide dehydratase activity
14.4	GO:0009701 Isoflavonoid phytoalexin biosynthetic process
12.6	GO:0102670 6a-hydroxymaackiain-3-O-methyltransferase activity
12.6	GO:0102671 2,7,4'-trihydroxyisoflavanone-4'-O-methyltransferase activity
9.9	GO:1990136 Linoleate 9S-lipoxygenase activity
9.9	GO:0030746 Isoflavone 4'-O-methyltransferase activity
9.7	GO:0005199 Structural constituent of cell wall
8.6	GO:0004866 Endopeptidase inhibitor activity
7.3	GO:0031408 Oxylipin biosynthetic process
6.7	GO:0034440 Lipid oxidation
4.1	GO:0004568 Chitinase activity
3.9	GO:0010951 Neg. reg. of endopeptidase activity
3.7	GO:0004364 Glutathione transferase activity
2.6	GO:0016311 Dephosphorylation
2	GO:0003700 Transcription factor activity-sequence-specific DNA binding

upf2

Fold	GO category
35.1	GO:0102670 6a-hydroxymaackiain-3-O-methyltransferase activity
35.1	GO:0102671 2,7,4'-trihydroxyisoflavanone-4'-O-methyltransferase..
34.4	GO:0009701 Isoflavonoid phytoalexin biosynthetic process
30	GO:0009715 Chalcone biosynthetic process
29.4	GO:0034007 S-linalool synthase activity
27.5	GO:0030746 Isoflavone 4'-O-methyltransferase activity
22.5	GO:0031347 Reg. of defense response
22	GO:2000022 Reg. of jasmonic acid mediated signaling pathway
17.5	GO:0004620 Phospholipase activity
17.3	GO:0004866 Endopeptidase inhibitor activity
16.5	GO:0030639 Polyketide biosynthetic process
15.2	GO:0009611 Response to wounding
14.6	GO:0016210 Naringenin-chalcone synthase activity
13	GO:0031408 Oxylipin biosynthetic process
13	GO:0034440 Lipid oxidation
10.1	GO:0010333 Terpene synthase activity
10.1	GO:0009813 Flavonoid biosynthetic process
7.8	GO:0016042 Lipid catabolic process
6.5	GO:0010951 Neg. reg. of endopeptidase activity
4.1	GO:0000287 Magnesium ion binding

Figure S6 GO enrichment analysis of the up-regulated differentially expressed genes (Up-DEGs) in *upf1*and *upf2* silenced roots. Comparison of top enriched Gene Ontology (GO) categories among Up-DEGs. GO categories enriched in both *upf1*and *upf2* silenced roots are highlighted with rectangles. Yellow and red rectangles indicate flavonoid biosynthesis-related and endopeptidase activity-related GOs, respectively. Fold indicates fold enrichment. Note that 8 out of the15 GO categories enriched in *upf1* are also enriched in *upf2*.

Enriched GOs of the Up-DEGs of the NMD core gene set

Enrichment	nGenes	Pathway genes	Fold	GO category
1.0E-03	3	4	44.5	GO:0004657 Proline dehydrogenase activity
9.4E-06	5	8	37.1	GO:0009701 Isoflavonoid phytoalexin biosynthetic process
2.1E-03	3	5	35.6	GO:0047987 Hydroperoxide dehydratase activity
2.1E-03	3	5	35.6	GO:0010133 Proline catabolic proc. to glutamate
2.0E-04	4	7	33.9	GO:0004737 Pyruvate decarboxylase activity
1.8E-06	6	11	32.3	GO:0102670 6a-hydroxymaackiain-3-O-methyltransf…
1.8E-06	6	11	32.3	GO:0102671 2,7,4'-trihydroxyisoflavanone-4'-O-methylt…
9.2E-06	6	14	25.4	GO:0030746 Isoflavone 4'-O-methyltransferase activity
7.8E-09	11	35	18.6	GO:0004866 Endopeptidase inhibitor activity
8.2E-05	6	21	16.9	GO:1990136 Linoleate 9S-lipoxygenase activity
2.0E-04	6	25	14.2	GO:2000022 Reg. of jasmonic acid mediated signaling pathway
1.4E-03	5	22	13.5	GO:0031347 Reg. of defense response
2.0E-05	8	38	12.5	GO:0031408 Oxylipin biosynthetic process
2.0E-05	8	38	12.5	GO:0034440 Lipid oxidation
8.0E-04	7	47	8.8	GO:0009611 Response to wounding
2.0E-04	9	71	7.5	GO:0016042 Lipid catabolic process
8.4E-07	15	119	7.5	GO:0010951 Neg. reg. of endopeptidase activity

Enriched GOs of the Down-DEGs of the NMD core gene set

Enrichment	nGenes	Pathway genes	Fold	GO category
1.6E-03	2	3	162	GO:0006809 Nitric oxide biosynthetic process
1.6E-03	2	3	162	GO:0050464 Nitrate reductase (NADPH) activity
3.4E-06	4	7	138.8	GO:0004737 Pyruvate decarboxylase activity
2.1E-04	3	7	104.1	GO:0051903 S-(hydroxymethyl)glutathione dehydrogenase activity
2.6E-04	3	8	91.1	GO:0009263 Deoxyribonucleotide biosynthetic process
4.8E-03	2	6	81	GO:0043546 Molybdopterin cofactor binding
3.1E-05	4	14	69.4	GO:0030976 Thiamine pyrophosphate binding
6.4E-04	3	11	66.3	GO:0004748 Ribonucleoside-diphosphate reductase activity-thioredoxin disulfide as acceptor
8.1E-03	2	8	60.7	GO:0009703 Nitrate reductase (NADH) activity
1.1E-03	3	14	52.1	GO:0004024 Alcohol dehydrogenase activity-zinc-dependent
1.6E-03	3	17	42.9	GO:0046294 Formaldehyde catabolic process
3.1E-03	3	23	31.7	GO:0005200 Structural constituent of cytoskeleton
4.3E-03	3	27	27	GO:0015250 Water channel activity
4.3E-03	3	27	27	GO:0006833 Water transport
1.1E-03	5	83	14.6	GO:0006334 Nucleosome assembly
4.6E-03	5	130	9.3	GO:0000786 Nucleosome
1.9E-03	6	164	8.9	GO:0045490 Pectin catabolic process

Figure S7 GO enrichment analysis of the up- and down-regulated differentially expressed genes (Up and down DEGs) of the NMD core gene set. Comparison of top enriched Gene Ontology (GO) categories (fold change >7). Yellow, red and green rectangles indicate GOs related to flavonoid biosynthesis, defense and nitrogen metabolism, respectively.

Analyzes of MAPK signaling KEGG pathway of *upf1* DEGs 1.

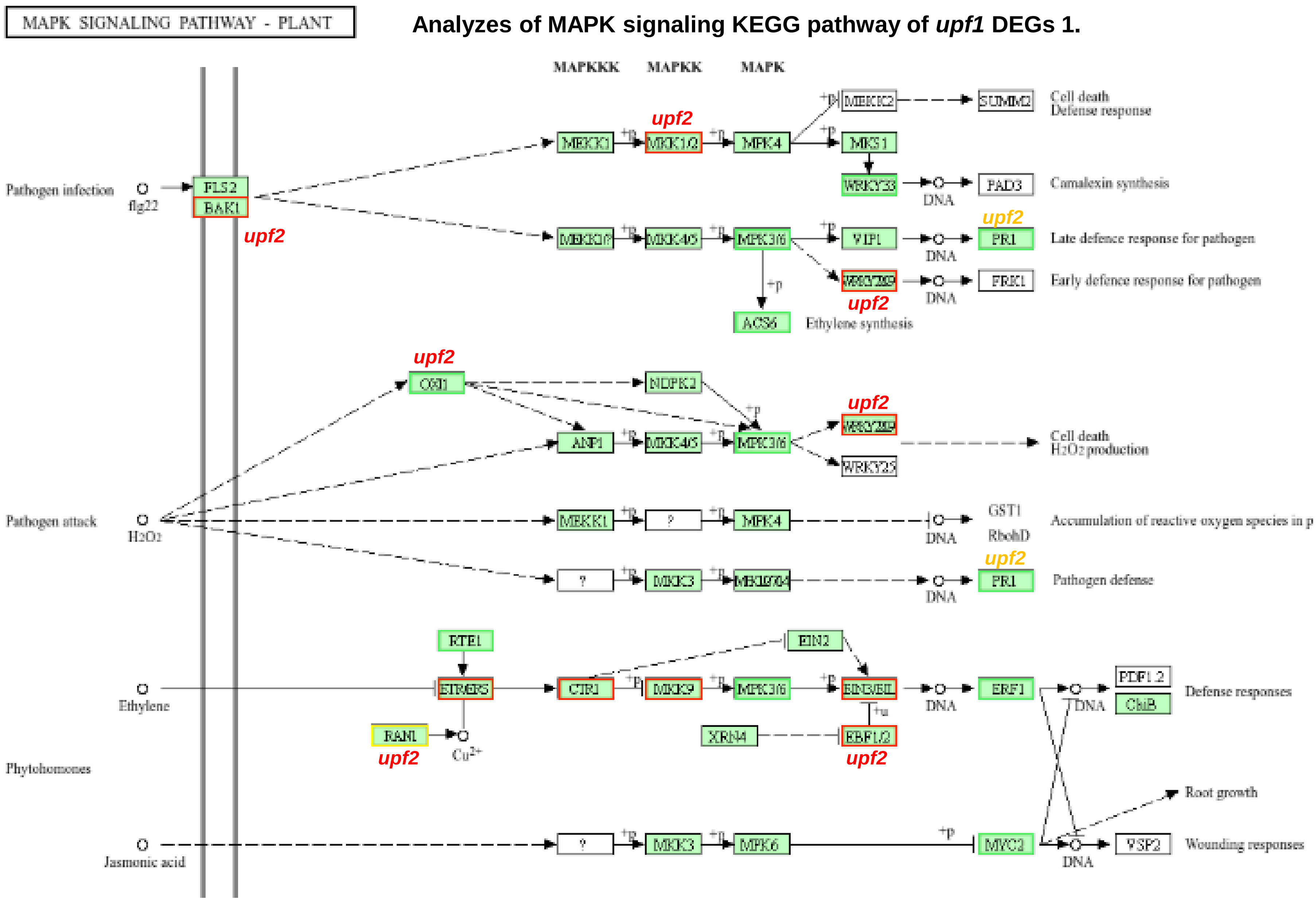

Figure S8 Analyzes of MAPK signaling KEGG pathway of *upf1* DEGs (continued at next slide)

Analyzes of MAPK signaling KEGG pathway of *upf1* DEGs 2.

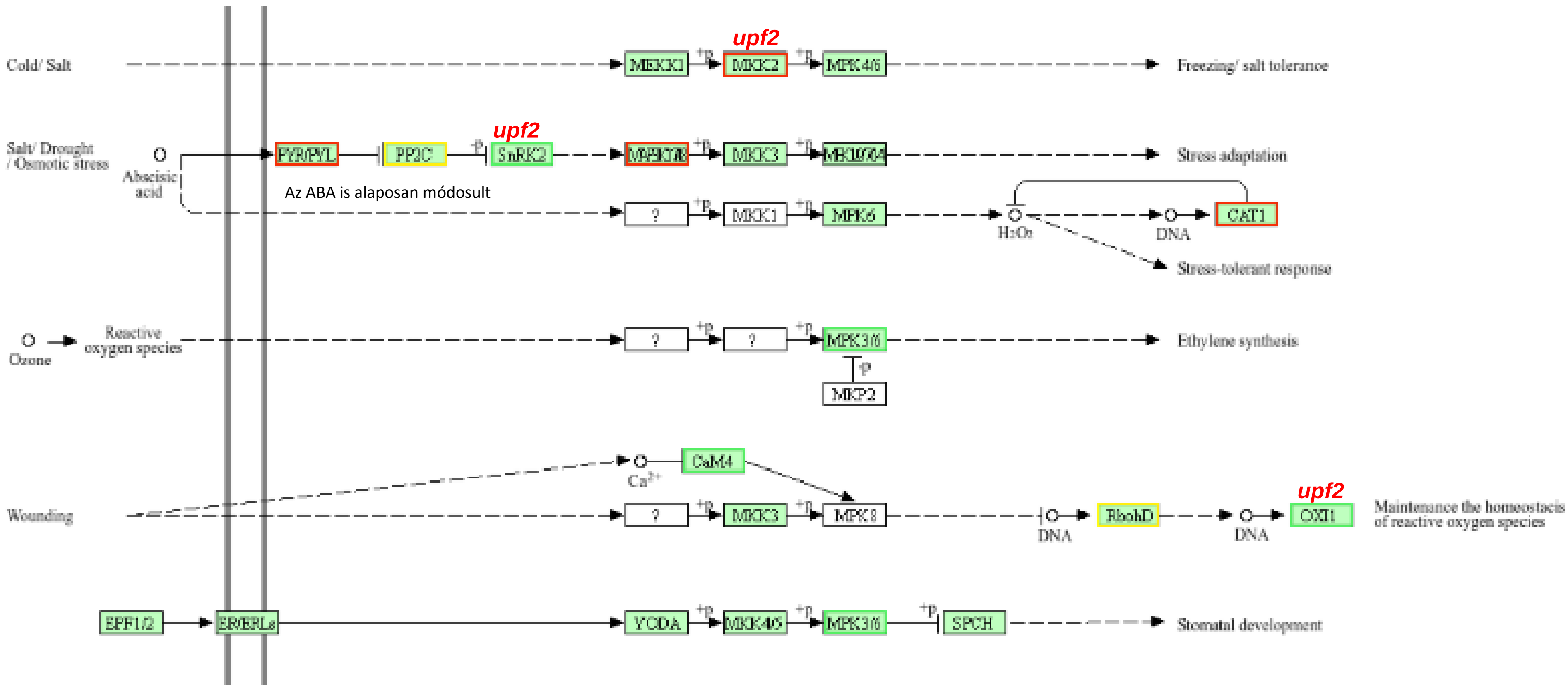

Figure S8 Analysis of the MAPK signaling KEGG pathway of *upf1* DEGs. Green boxes indicate pathway components that can be identified in *Medicago truncatula*. A given box may represent a single gene, if the factor is present as a single copy in *M. truncatula*, or multiple genes, if it is encoded by more than one gene copy. Black-framed boxes show components whose corresponding genes were not differentially expressed in *upf1*. Red framed boxes indicate components for which the corresponding gene is an upregulated DEG (Up-DEG) in *upf1* or in the case of multicopy genes, at least one copy is an Up-DEG, while the others show no significant change. Green-framed boxes indicate that at least one gene copy is downregulated (Down-DEG), and yellow-framed boxes indicate that both Up- and Down-DEGs are present. Red, green and yellow labeled characters for *upf2* indicate if the corresponding factor is an Up-DEG, a Down-DEG, or shows mixed regulation in *upf2*. Notably, although fewer components are altered in *upf2*, most affected components show similar expression changes in both *upf1* and *upf2* RNAi roots.

Analyzes of Nitrogen Metabolism KEGG pathway of *upf1* DEGs

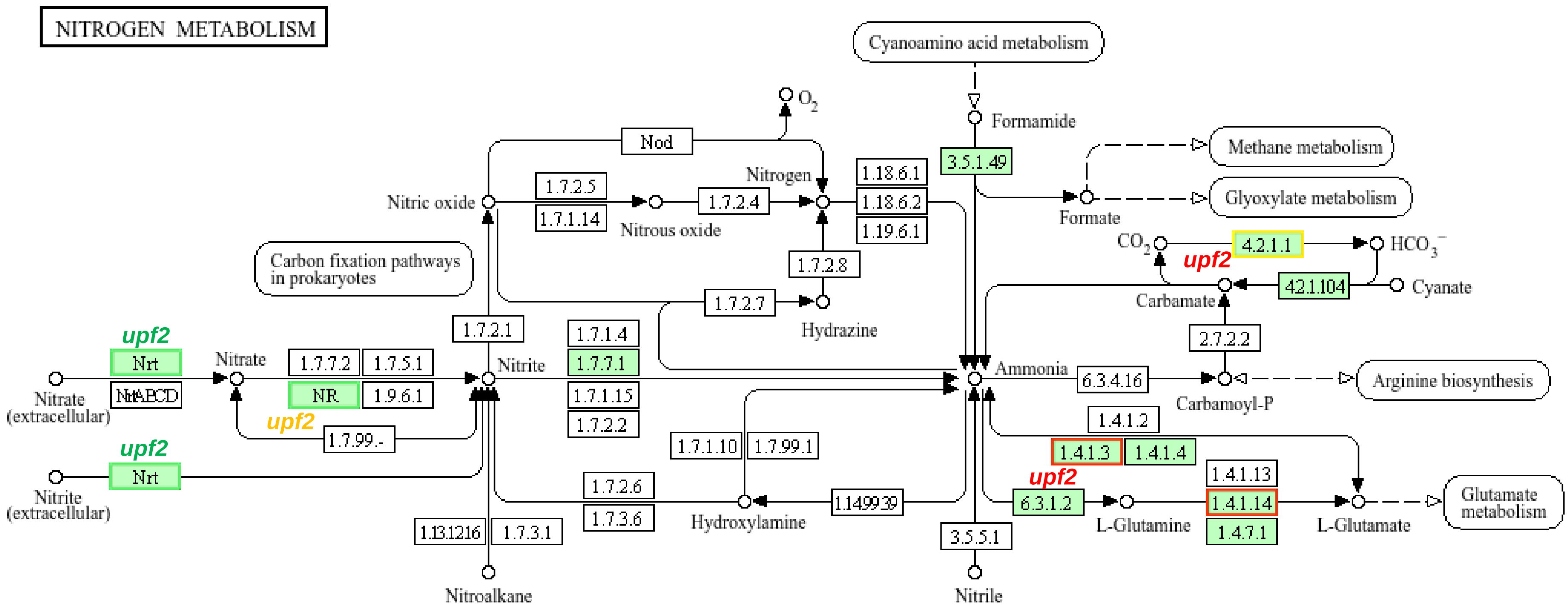

Figure S9 Analyzes of Nitrogen Metabolism KEGG pathway of *upf1* DEGs. Symbols are described at Figure S7.

Analyzes of Flavonoid biosynthesis KEGG pathway of *upf1* DEGs

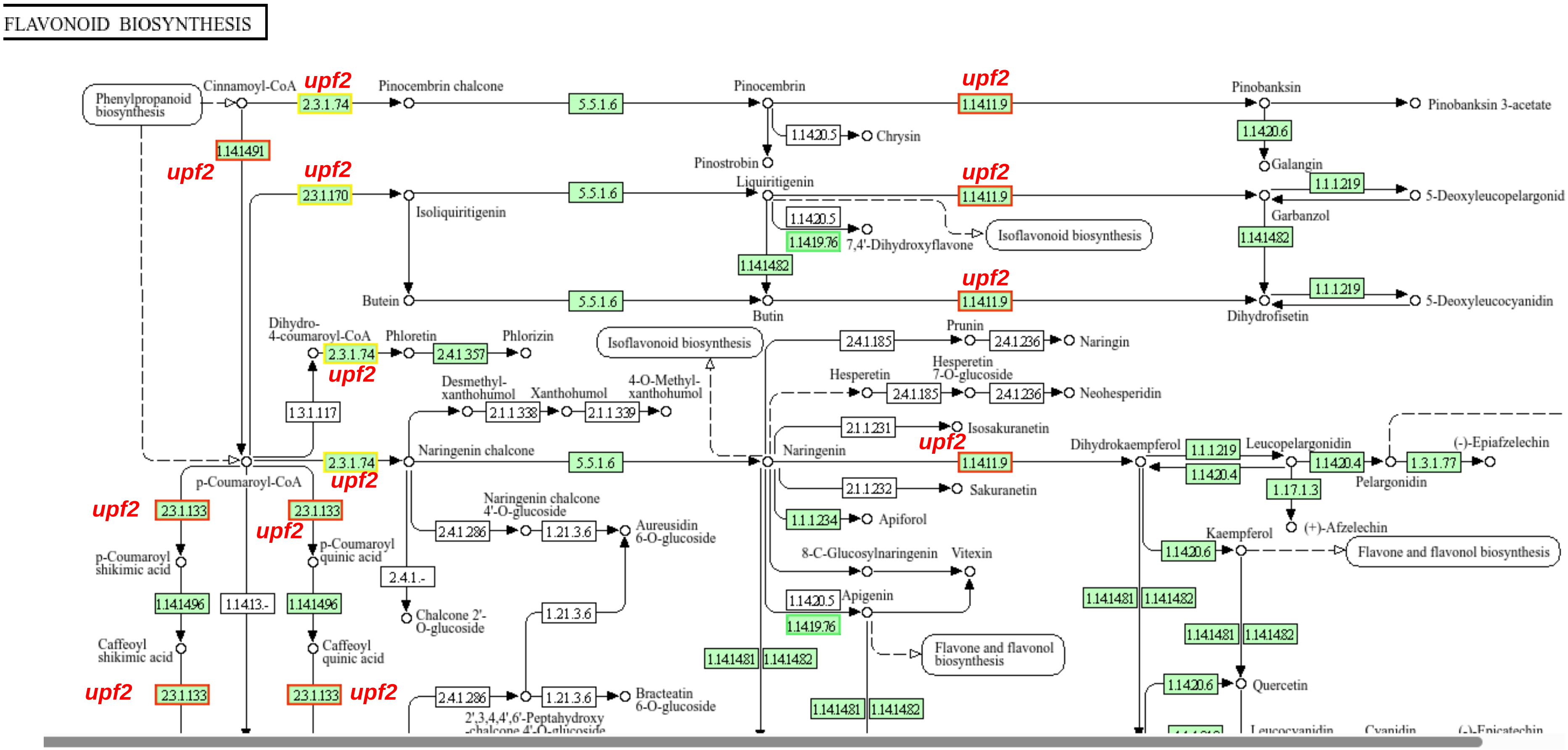

Figure S10 Analyzes of Flavonoid biosynthesis KEGG pathway of *upf1* DEGs. Symbols are described at Figure S7.

Analyzes of Linoleic Acid metabolism KEGG pathway of *upf1* DEGs

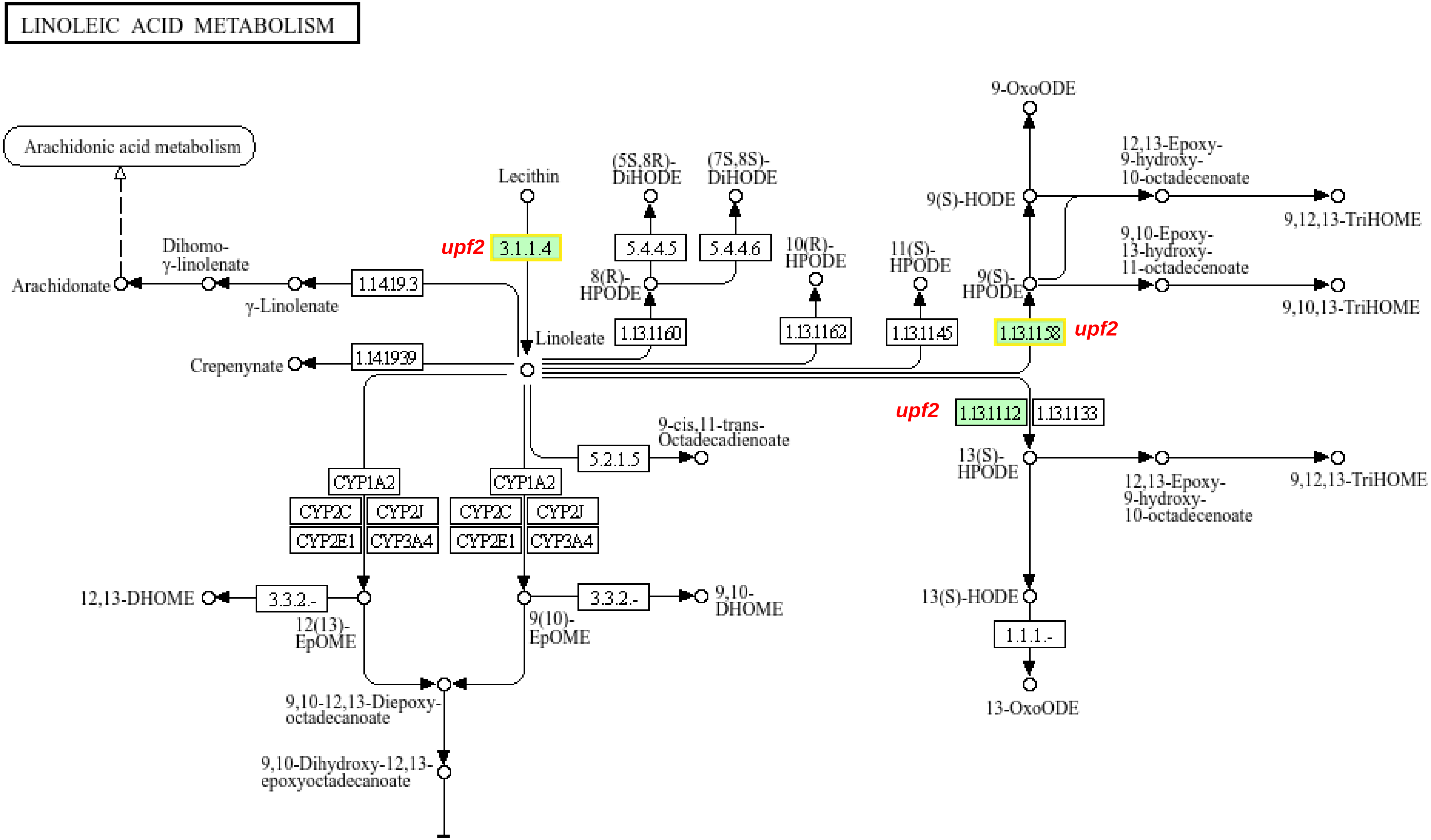

Figure S11 Analyzes of Linoleic Acid metabolism KEGG pathway of *upf1* DEGs. Symbols are described at Figure S7.

Analyzes of Alfa Linoleic Acid metabolism KEGG pathway of *upf1* DEGs

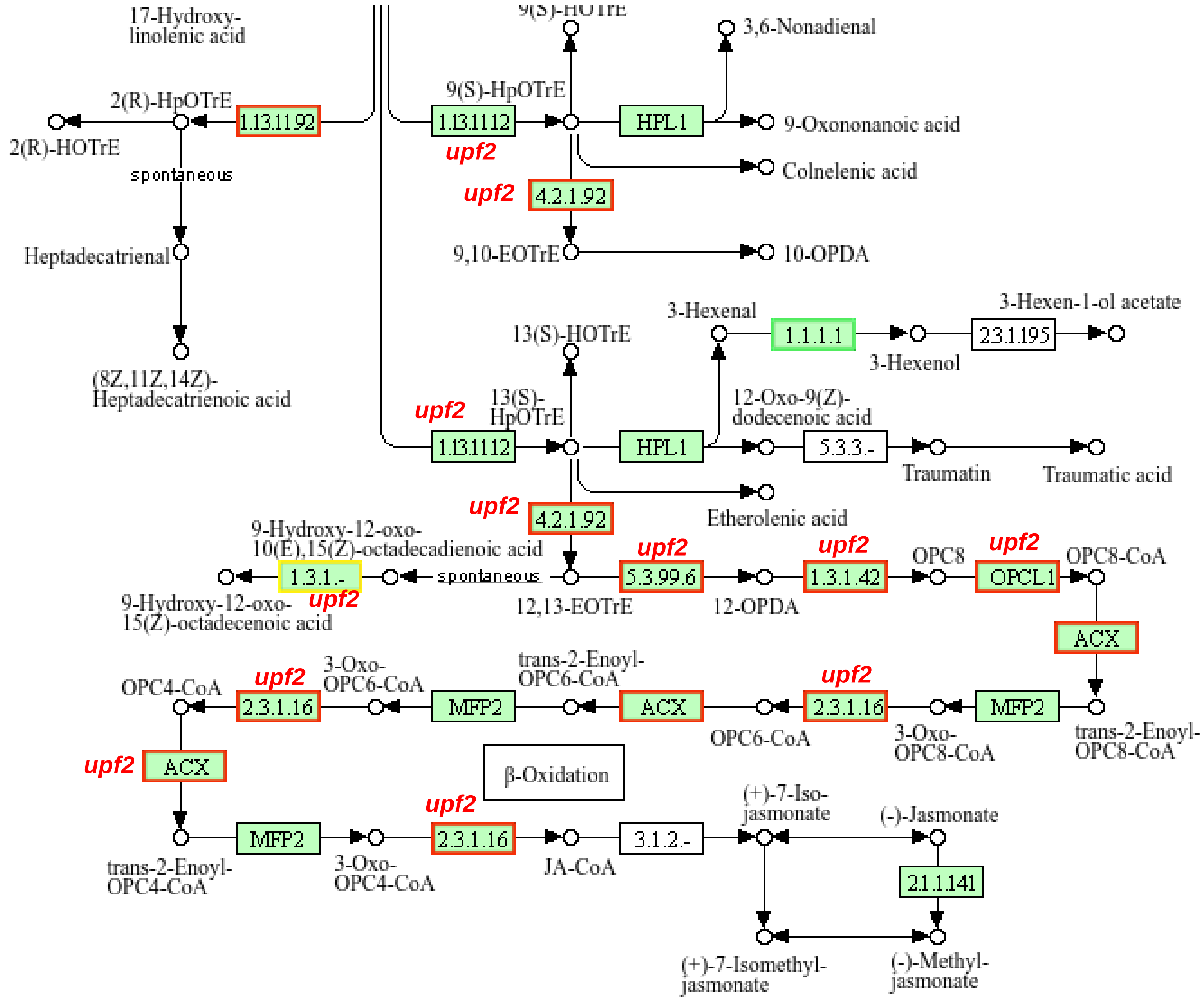

Figure S12 Analyzes of Alfa Linoleic Acid metabolism KEGG pathway of *upf1* DEGs. Symbols are described at Figure S7.

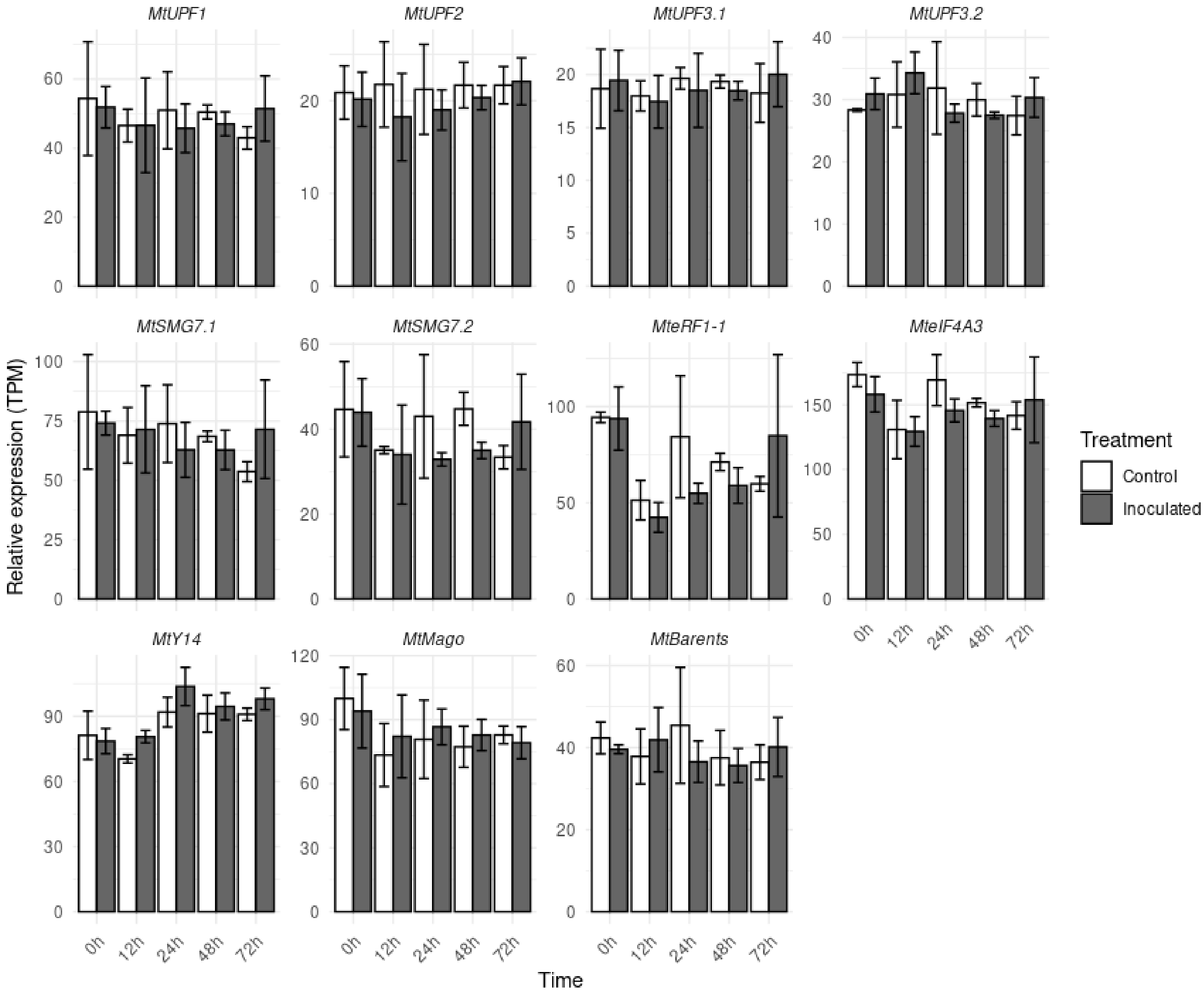

Figure S13 Expression of NMD factor genes during the early phase of nodulation. Raw sequencing data were retrieved from the SRA database (NCBI Bioproject ID: PRJNA524899) and analyzed as described in the Materials and Methods section. Roots of *M. truncatula* plants were inoculated either with mock (Control) or *S. medicae* bacteria (Inoculated) and samples were collected at the indicated time points in three biological replicates. Relative expression values are in Transcript per Million (TPM) units. Error bars represent standard deviation. The results of two-way ANOVA tests are shown in the table.

(a)

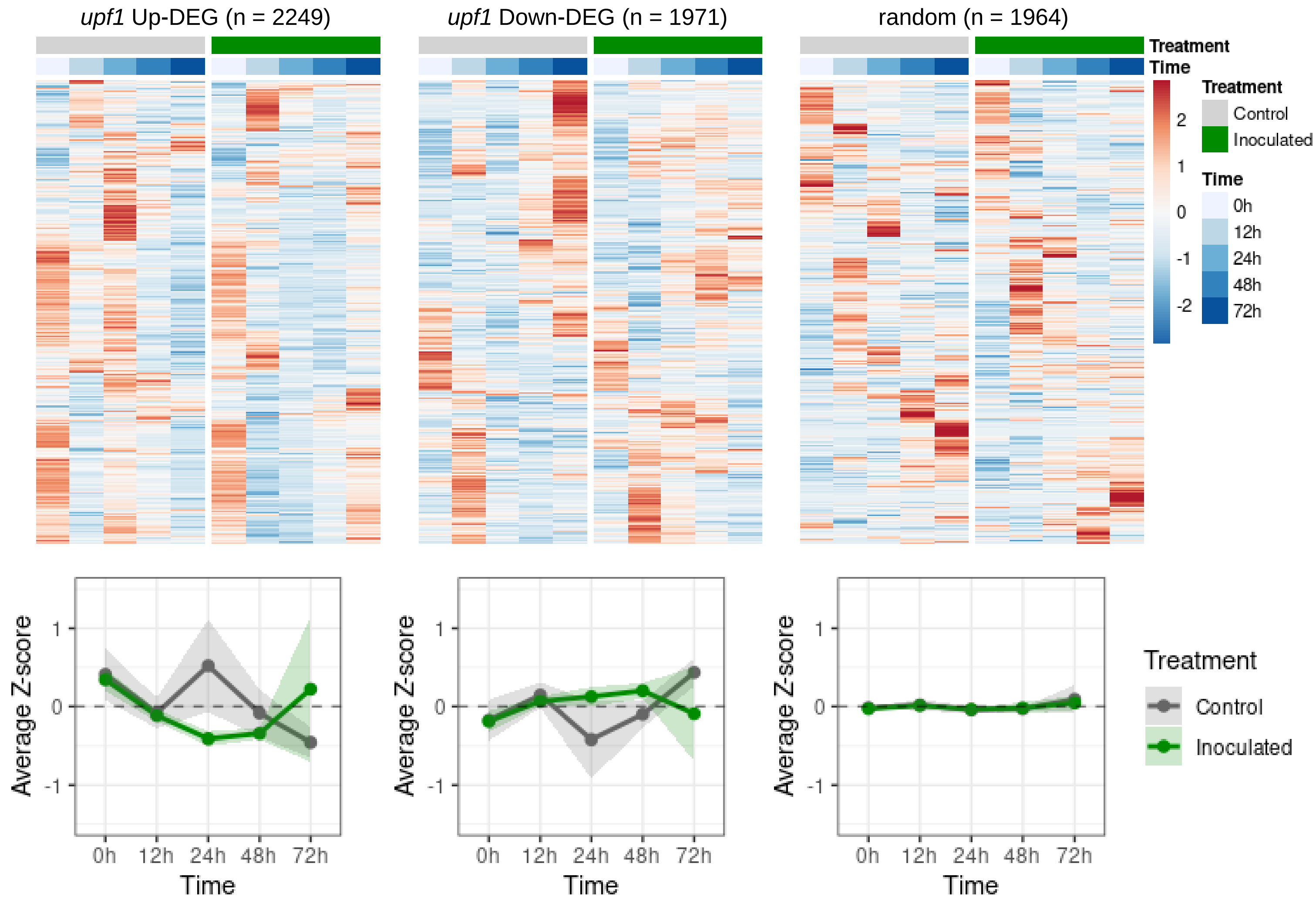

Figure S14 Expression of *upf1* DEGs during the early phase of root nodule symbiosis (RNS). (a) The dataset used is the same as in Figure S13. In the upper panel, the expression profiles of genes differentially expressed (DEGs) in *upf1* RNAi roots are shown. Upregulated and downregulated genes (Up-DEG and Down-DEG, respectively), along with 2,000 randomly selected genes are displayed as heatmaps (genes not expressed in this dataset were excluded). Colors represent Z-scores. In the lower panel, the mean Z-score of the genes shown in the heatmaps were calculated and plotted to assess overall expression trends within each gene set. Shaded areas represent standard deviation. Contrary to the hypothesis that NMD is inhibited during nodulation, an effect that would be expected to lead to accumulation of NMD targets, the *upf1* Up-DEGs exhibited a slight downward trend in inoculated roots at 24h post-inoculation compared with mock-treated controls. The randomly selected genes did not display any consistent pattern.

(b)

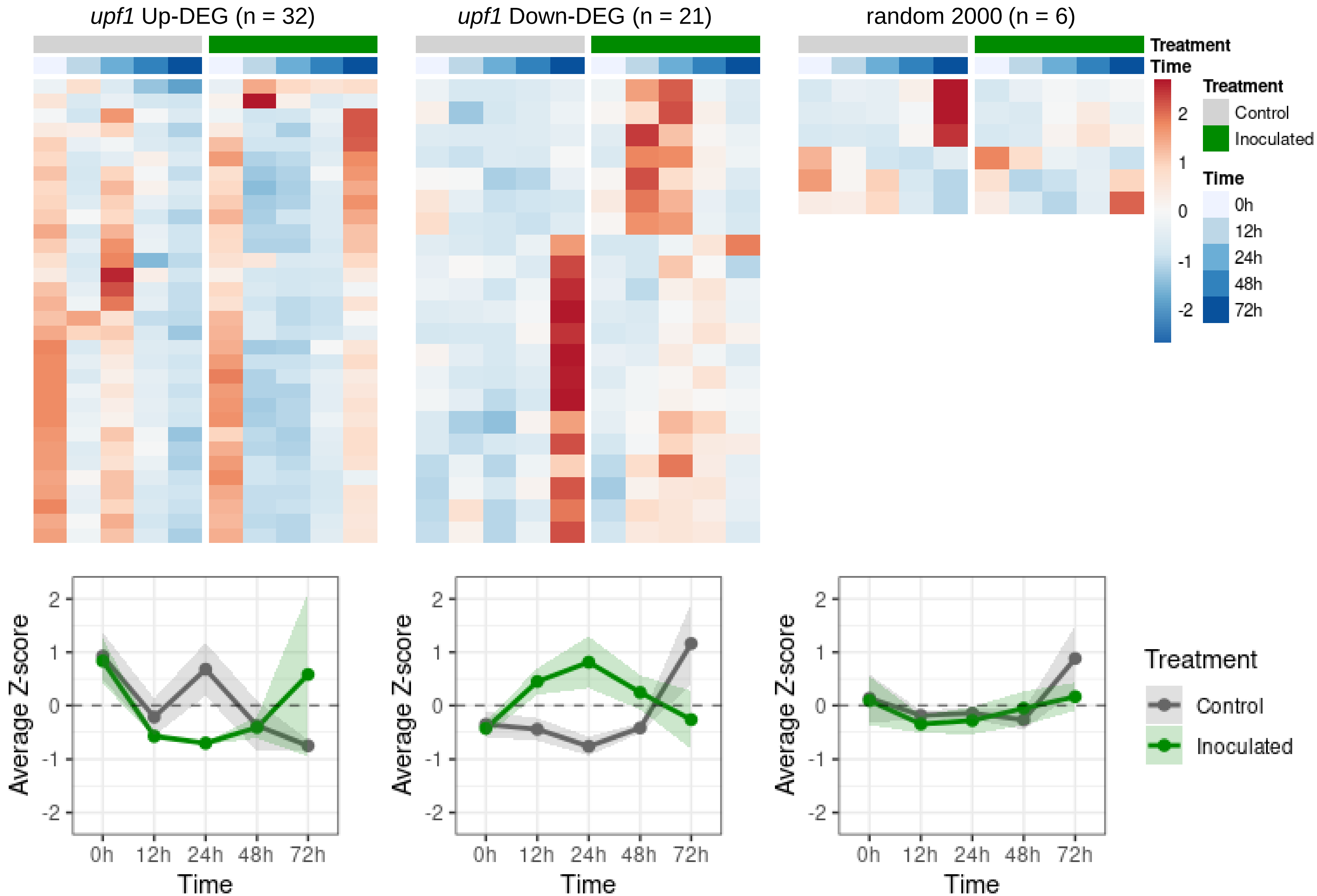

Figure S14 Expression of *upf1* DEGs during the early phase of root nodule symbiosis (RNS). (b) As is panel (a) but restricted to genes whose expression is significantly affected by the interaction between Time and Treatment. To identify genes specifically responding to inoculation over time, a Likelihood Ratio Test was applied. Only a small subset of *upf1* DEGs (32 Up and 21 Down) exhibited significant expression changes. The more pronounced opposing trends observed in these filtered sets further support the conclusion that NMD remains active,or even be enhanced, during the early stages of nodulation.

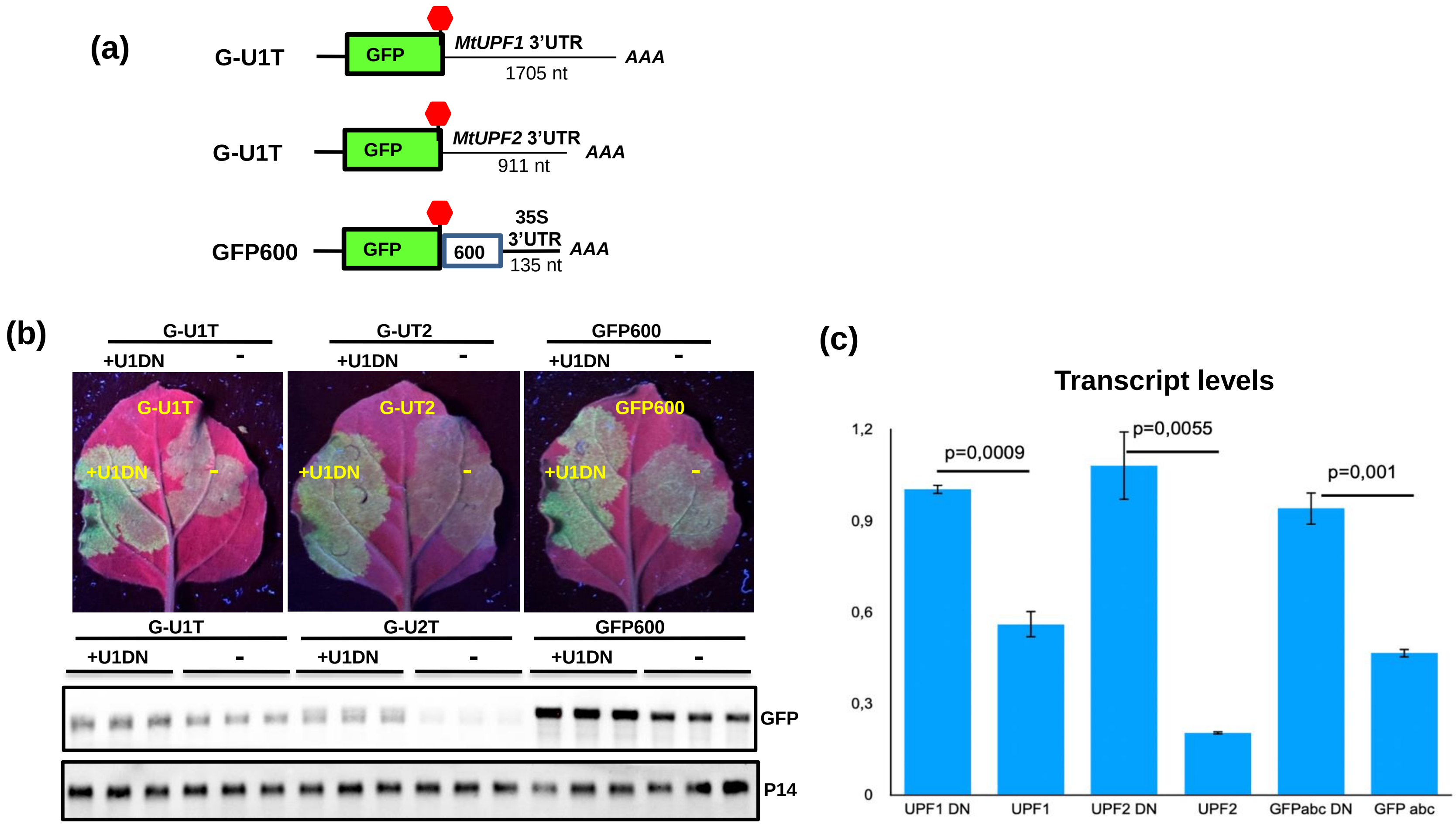

Figure S15 The 3'UTRs of *MtUPF1* and *MtUPF2* induce NMD. (a) Schematic, non-proportional representation of reporter constructs. The terminator regions of *MtUPF1* and *MtUPF2* were cloned downstream of a GFP reporter (G-U1T and G-U2T). (b) These constructs were agroinfiltrated into *Nicotiana benthamiana* leaves in the presence (U1DN) or absence (-) of the dominant-negative version of *Arabidopsis* UPF1. The P14 silencing suppressor was co-infiltrated and used as normalization control. GFP600, a previously described NMD sensitive reporter, was used as positive control. Photographs were taken and RNA samples were collected at 3 days post-infiltration. RNA gel blots were hybridized with P14 and GFP probes. (c) For quantification, the reporter mRNA signal (GFP probe) in each lane was normalized to the corresponding P14 signal (GFP/P14 signal). Mean values were calculated from three independent replicates. The GFP/P14 ratio of the U1DN co-infiltrated sample was set to 1, and the GFP/P14 ratio of the (-) sample is shown relative to this value. Error bars represent standard deviation. Student's t-test was used to assess significance.

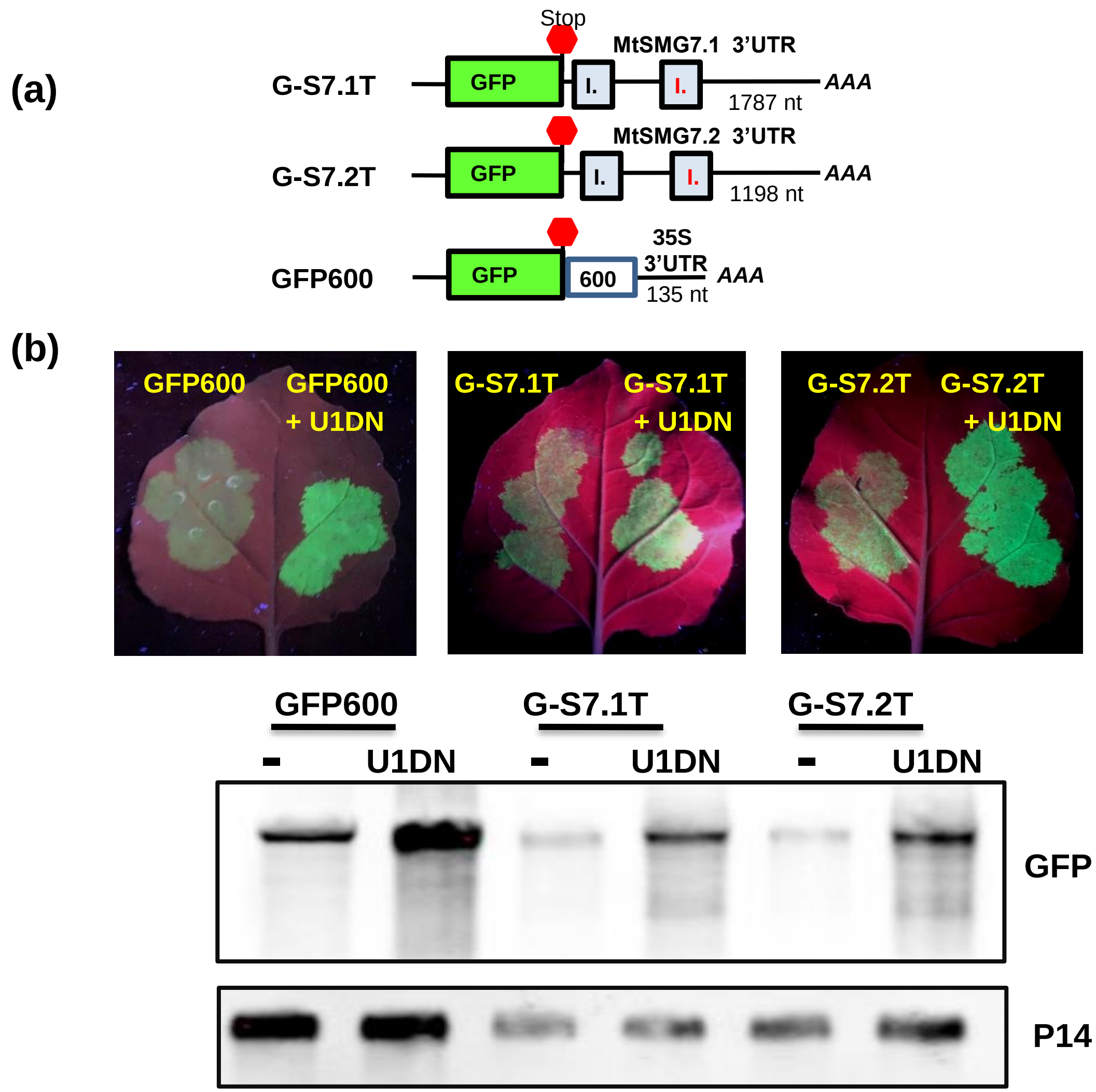

Figure S16 The 3'UTR of *MtSMG7.1* and *MtSMG7.2* activate NMD. (a) The terminator regions of *MtSMG7.1* and *MtSMG7.2* were cloned downstream of a GFP reporter (G-S7.1T and G-S7.2T). Introns (I.) located in the 3'UTR are indicated with black I. and red I. letters represent non-NMD relevant (located < 50 nt from the stop codon) and NMD-relevant introns (> 50 nt from the stop codon) introns, respectively. (b) The reporter constructs were agroinfiltrated into *Nicotiana benthamiana* leaves in the presence (U1DN) or absence (-) of the dominant-negative version of *Arabidopsis* UPF1. GFP600, a previously described NMD sensitive reporter, was used as a positive control. The P14 silencing suppressor was co-infiltrated and used as normalization control. Note that both 3'UTR were sensitive to NMD although the *SMG7.1* terminator showed relatively low efficiency in *N. benthamiana*. RNA gel blots were hybridized with P14 and GFP probes to assess RNA abundance.
