# Supplementary material for: Nonsense‐Mediated Decay mRNA Quality Control System Is Essential for Root Development and Efficient Root Nodule Symbiosis in Medicago truncatula
Source: Plant Cell Environ. 2026 Jun 21;49(9):7210–4. doi: 10.1111/pce.70683 (PMC13436451; doi:10.1111/pce.70683)
Supplement: Supplementary file 2 [file PCE-49-7210-s006.docx]

**Table S1.** The putative *M. truncatula* NMD factors

.

| **Gene** | **Arabidopsis** | ***M. truncatula* (annotation 5)** | **NMD relevant features** | | |
| --- | --- | --- | --- | --- | --- |
|  |  |  | **5’UTR**  **(uORF<30 aa)** | **3’UTR** | |
|  |  |  |  | **Long 3’UTR**  **(<500 nt)** | **NMD-relevant intron**  **(< 50 nt from the stop)** |
| **NMD core factors** | | | | | |
| ***UPF1*** | AT5G47010 | **MtrunA17_Chr5g0400101** | No | **1705 nt** | No |
| ***UPF2*** | AT2G39260 | **MtrunA17_Chr2g0303641** | No | **911 nt** | No |
| ***UPF3.1*** | AT1G33980 | MtrunA17_Chr3g0077481 | No | **1138** | 2 introns:  9 nt non-NMD  **142 nt NMD-relevant** |
| ***UPF3.2*** |  | [MtrunA17_Chr2g0276971](https://medicago.toulouse.inra.fr/MtrunA17r5.0-ANR/web/fatal/entry.html?accession=MtrunA17_Chr2g0276971) | **31** | **759** | No  (13 nt not relevant) |
|  | | | | | |
| ***SMG7.1*** | AT5G19400 | MtrunA17_Chr5g0441821 | No | 1787 | 2 introns:  22 nt non-NMD  **125 nt NMD-relevant** |
| ***SMG7.2*** |  | [MtrunA17_Chr3g0094441](https://medicago.toulouse.inra.fr/MtrunA17r5.0-ANR/web/fatal/entry.html?accession=MtrunA17_Chr3g0094441) | No | 1198 | 2 introns:  31 nt not-NMD  **155 nt NMD-relevant** |
| **eRF1-1 translation termination factor** | | | | | |
| ***eRF1-1*** | AT5G47880 | MtrunA17_Chr6g0481411 | No | **764** | 4 introns:  **152 nt NMD-relevant**  **286 nt NMD-relevant 366 nt NMD-relevant**  **442 nt NMD-relevant** |
| **EJC factors** | | | | | |
| ***eIF4A3*** | At3g19760 | MtrunA17_Chr2g0297261 | no | **888** | no |
| ***Y14*** | **AT1G51510** | MtrunA17_Chr5g0396371 | no | **901** | no |
| ***Mago*** | [AT1G02140](https://bar.utoronto.ca/thalemine/report.do?id=4010942&trail=%7c4010942) | MtrunA17_Chr3g0145771 | no | **1003** | no |
| ***Barents*** | At1g80000 | [MtrunA17_Chr4g0004221](https://medicago.toulouse.inra.fr/MtrunA17r5.0-ANR/web/fatal/entry.html?accession=MtrunA17_Chr4g0004221) | no | **729** | 3 introns:  42 nt non-NMD  **118 nt NMD-relevant**  **201 nt NMD-relevant** |
