# Supplementary material for: Nonsense‐Mediated Decay mRNA Quality Control System Is Essential for Root Development and Efficient Root Nodule Symbiosis in Medicago truncatula
Source: Plant Cell Environ. 2026 Jun 21;49(9):7210–4. doi: 10.1111/pce.70683 (PMC13436451; doi:10.1111/pce.70683)
Supplement: Supplementary file 3 [file PCE-49-7210-s003.doc]

**Supporting materials**

**Supplementary Discussion**

Here, we have demonstrated that the NMD mRNA quality control system plays a critical role in root development and nitrogen-fixing symbiotic interaction in *M. truncatula*. Complete inactivation of NMD was not achieved, as knockout of the core factors *MtUPF1* or *MtUPF2* was unsuccessful (Figure S1), suggesting that NMD is essential for root viability. (Suppl. Figure 1). However, partial NMD impairment was achieved using *upf1* and *upf2* RNAi roots. These NMD-deficient roots developed normally but showed altered expression of numerous RNS-related genes and pathways and formed ineffective symbiotic interactions (Figure 1). Although the NMD inhibition was less efficient in the *upf2*, the infection and nodulation were comparably impaired in *upf1* and *upf2* roots, indicating that root nodule symbiosis (RNS) is sensitive to NMD activity. Even moderate NMD reduction markedly reduces symbiotic efficiency. Our results show that efficient NMD is required for the symbiotic competence of the roots (Figure 1), and suggest that the NMD activity remains unchanged during early phase of RNS (Suppl. Figure 9-10).

**NMD is required for root development in *Medicago truncatula***

To elucidate the role of NMD in RNS, we aimed to generate knock out (KO) mutants of *MtUPF1* and *MtUPF2*, the most conserved and important NMD components in *M. truncatula* roots, and then assess the symbiotic competence of the KO roots. We have previously used CRISPR/Cas9 gene editing system successfully to knock-out several nodulation-specific genes; and in each case, identified numerous nodules in which 100% of the alleles were edited (Güngör et al., 2023; Saifi et al., 2024). However, we were unable to generate homozygous *MtUPF1* or *MtUPF2* KO roots (Suppl. Figure 2).

For *MtUPF1*, we did not identify mutant roots in which more than 67% of the sequenced alleles carried frameshift mutations. The remaining alleles were either wild-type or likely functional mutant variants containing short in-frame deletions. Similarly, we failed to obtain homozygous *MtUPF2* KO roots; fewer than 68% of the alleles carried frameshift mutations, while the remaining alleles were all wild-type (Suppl. Figure 2). These results suggest that *MtUPF2* mutants harboring short in-frame deletions in this region are non-functional. Relevantly, although KO mutants could be generated for multiple nodulation factors, this was not possible for either *MtUPF1* or *MtUPF2* NMD components, indicating that NMD is essential for root development in *M. truncatula*.

As KO mutants could not be obtained, we next attempted to reduce *MtUPF1* and *MtUPF2* expression using RNA interference (RNAi) approach. Silencing was selective: in *upf1* and *upf2* RNAi roots, the mRNA levels of the targeted NMD factor were reduced to ~40-50% relative to the control, while the expression of the non-targeted factor were not decreased and was, in fact, elevated. The relatively modest silencing efficiency of NMD factors is consistent with the hypothesis that NMD is essential for root growth. We propose that transgenic roots, in which NMD is very efficiently inactivated, fail to develop. Together, the inability to generate NMD KO roots and the limited reduction of NMD factor expression achieved by RNAi strongly support the conclusion that NMD activity is required for root development.

Although transcript levels of the target genes were only moderately reduced and the roots growth appeared normal, RT-qPCR and sequencing results confirmed that NMD was impaired in the RNAi roots. It may seem surprising that a 40-50% reduction in mRNA leads is sufficient to induce NMD-deficiency, given that heterozygous NMD mutants show wild-type phenotype. However, because siRNAs can also inhibit translation (Brodersen et al., 2008), the reduction in UPF1 and UPF2 protein levels may be more pronounced than suggested by transcript abundance alone. Unfortunately, in the absence of specific antibodies, we were unable to directly determine UPF1 and UPF2 protein levels in the RNAi roots.

**The impact of NMD-deficiency on root nodule symbiosis**

NMD regulates large numbers of genes in *M. truncatula*; with more than 15% of the genes were identified as differentially expressed genes (DEGs) in *upf1* roots. Moreover, it is likely that several weak NMD targets were not classified as DEG, for instance, the potential direct NMD target *UPF2* was up-regulated by less than twofold. Early rhizobia infection events were strongly reduced in both *upf* silenced roots, indicating that NMD-deficiency severely compromises the symbiotic competence of the root (Figure 1).

How does NMD-deficiency inhibit early rhizobia interactions? Because NMD-deficient roots grow normally, it is unlikely to explain the observed symbiotic defects. In *Arabidopsis*, NMD-deficiency results in the overexpression of resistance (R)-genes and autoimmunity (Gloggnitzer et al., 2014; Rayson et al., 2012). Although it is tempting to speculate that the upregulation of R-genes might hinder symbiosis in *upf* roots, the putative *M. truncatula* R-genes (Calle García et al., 2022; Zhai et al., 2011) were not overrepresented among the DEGs in either *upf1* or *upf2* mutant roots (Table S4). Instead, we found that many RNS-related pathways are perturbed in NMD-deficient roots (Figures S7-11). Given that efficient symbiosis requires tightly coordinated host-microbe interactions, even modest disruptions in RNS-critical genes and pathways likely impair infection efficiency.

Several pathways that may contribute to reduced symbiotic competence are altered in NMD-deficient roots (Figures S7-11). Here, we briefly discuss how modifications of different RNS-related pathways in NMD-deficient roots may lead to impaired RNS.

MAPK signaling, plant hormone signal transduction and plant-pathogen interaction pathways are interconnected and significantly modified in NMD-deficient roots (Figure S3). The components of the pathogen response pathway, including BAK1, MAPKK1/2 and WRKY29 are up-regulated (Figure S7), suggesting priming of defense responses, that may inhibit early symbiotic interactions. BAK1, the central co-receptor of multiple cell surface receptors including the Nod factor-receptor LjNFR5 and the symbiosis receptor LjSymRK/MtDMI2 (Feng et al., 2021; Tsikou et al., 2018), is known to balance growth and defense (Y. Wu et al., 2025). The overexpression of *Bak1* inhibits nodulation in *Lotus japonicus* (Feng et al., 2021), indicating that BAK1 up-regulation may contribute to the reduced symbiotic efficiency of NMD-deficient roots. Although the role of MAPKK1/2 overexpression on nodulation has not been directly examined, MAPK signaling generally acts as a negative regulator of symbiosis (Ryu et al., 2017). In *Arabidopsis*, increased WRKY29 expression, together with altered ethylene signaling components, further supports enhanced defense signaling (Asai et al., 2002; Wang et al., 2023). Because ethylene negatively regulates symbiosis (Varma Penmetsa et al., 2008), its elevated signaling may suppress infections, although concurrent upregulation of EIN3-binding F-box protein genes EBF1 and EBF2, which are also enhanced in the NMD-deficient roots, suggests complex regulation of this pathway.

The *Oxi1* (*MtSpk1*) gene is a key component of reactive oxygen species (ROS) signaling, which plays a critical early role in symbiosis. *MtSpk1* expression is significantly modified in the roots of both RNAi lines, being down-regulated in *upf1* and up-regulated in *upf2*. Because *MtSpk1* inactivation impairs symbiosis (Andrio et al., 2013), while its overexpression activates defense responses in *Arabidopsis* (Rawat et al., 2023), proper *MtSpk1* expression levels appear to be critical for efficient symbiosis.

Nitrogen metabolism pathway is also disrupted, as nitrate reductases *NR1* and *NR2* and the nitrate transporter *NRT2.1* are also significantly downregulated in both NMD-deficient roots (Figures S6 and S8). Because nodulation is almost completely abolished in the *nr1/nr2* double mutant (<https://www.biorxiv.org/content/10.1101/2024.05.31.596865v1>) and *NRT2.1* is required for optimal nodulation at low nitrogen condition (Luo et al., 2023), reduced expression of these genes may further compromise symbiosis.

The flavonoid and isoflavonoid biosynthetic pathways (Figures S3 and S9) are also strongly affected. These metabolites are key signaling molecules in symbiosis, and their altered production may impair early signaling and contribute to reduced infection frequencies in NMD-deficient roots. As some isoflavonoids function as phytoalexins, their altered levels may additionally enhance defense responses and inhibit symbiosis (W. Wu et al., 2024; Yang & Wang, 2024).

Finally, several components of the linoleic and alpha-linoleic acid pathways (Figures S3 and S10-11) are up-regulated in NMD-deficient roots, suggesting increased production of oxylipins, including jasmonic acid. Because jasmonic acid, which is synthesized from alpha-linoleic acid and negatively regulates symbiosis at high levels (Raza et al., 2021; Sun et al., 2006), as well as other oxygenated derivatives (oxylipins) of these unsaturated fatty acids are involved in signaling and defense (Sugimoto et al., 2022), may further contribute to impaired symbiosis.

Taken together, these finding indicate that the NMD quality control system regulates the expression of multiple factors critical for rhizobia symbiosis in *M. truncatula*, providing an explanation for severely reduced symbiosis efficiency in NMD-deficient roots. Further studies are required to elucidate the precise molecular basis of this impairment and to determine whether additional mRNA and protein quality control systems are also required for efficient nitrogen-fixing symbiosis.

**NMD activity is regulated by multiple autoregulatory circuits in *M. truncatula***

Despite weaker NMD-impairment in *upf2*, demonstrated by target mRNA reduction and less efficient induction of NMD reporter transcripts, fewer DEGs, less significantly enriched common KEGG pathways in *upf2* than in *upf1*), *upf2* and *upf1* RNAi roots showed similarly reduced symbiotic competence (Figure 1 and Figure S3), suggesting that NMD activity must be tightly controlled. Indeed, transcripts encoding several NMD components (*MtUPF1–3, MtSMG7.1, MtSMG7.2, MteRF1-1,* and *MtBarentsz*) are upregulated in NMD-deficient roots, indicating the presence of multiple negative feedback loops.

*SMG7* and *eRF1-1* mRNAs are conserved direct NMD targets with long 3’UTR containing NMD-relevant introns in all angiosperms. We previously proposed that the SMG7 and eRF1-1 autoregulatory circuits play a dominant role in stabilizing NMD activity in seed plants (Nyikó et al., 2013, 2017). Consistent with previous results, *MtSMG7.1*, *MtSMG7.2* and *MteRF1-1* mRNAs were significantly overexpressed in NMD-deficient *M. truncatula* roots. Moreover, both *MtSMG7* transcripts are direct NMD targets (Figure 1 and Figure S15).

The transcripts of *MtUPF1*, *MtUPF2* and *MtUPF3* were also upregulated in NMD-deficient roots, indicating that all four NMD core components are negatively regulated by NMD in *M. truncatula*. *MtUPF1* and *MtUPF2* appear to be weak NMD targets, as their transcripts show modest upregulation in NMD-deficient roots despite their NMD sensitive 3’UTRs in *N. benthamiana* leaves (Figure S14). The regulation of *MtUPF3.2* is less clear, as its 3’UTR does not activate NMD. In *Arabidopsis*, *UPF3* transcripts are direct NMD targets as they contain NMD-relevant uORFs (Degtiar et al., 2015; Vexler et al., 2016). The *MtUPF3.2* mRNA also contains a 30-amino acid long uORF, which may activate NMD in *M. truncatula*. Alternatively, *MtUPF3.2* may be an indirect NMD target.

Barentsz is the only exon junction complex (EJC) component regulated by NMD in *Arabidopsis* as well as in *M. truncatula*, and *Barentsz* transcripts contain NMD-relevant introns in the 3’UTR in both plants (Table S1). Barentsz repression by the intron-based NMD suggests a conserved regulatory circuit in which intron-based NMD regulates *Barentsz* mRNA levels, thereby controlling EJC expression and, in turn intron-based NMD (Nyikó et al., 2013).

Although reduced NMD activity impairs RNS, assessing the effects of increased NMD is challenging due to unknown limiting NMD components and multiple negative feedback mechanisms in *M. truncatula*.

In summary, NMD activity in *M. truncatula* roots is finely tuned my multiple autoregulatory circuits, and proper NMD function is essential for efficient nitrogen-fixing symbiosis.

**Supplementary Materials and Methods**

**List of primers used in this study**

| **Name of the primer** | **Sequence** |
| --- | --- |
| **Primers used for terminator assays** |  |
| BI MtUPF1 term F | TCCT*GGATCC*GGAACTTTAGAAGCTTAATGTGGCTT |
| EI MtUPF1 term short R | CAAC*GAATTC*TTGGGTGTGACGACTACCAAGAGGT |
| BI MtUPF2term F | CAAC*GGATCC*ATGATACTTGAGTGAATGTGGAAGGCA |
| EI MtUPF2 term R | CAAC*GAATTC*ACCCACTTAATGGTATGACCCACAA |
| Mt UPF3 Ter BI For | CAAC*GGATCC*AGTATTTAGATTGGTGAGACACATTCC |
| Mt UPF3 Ter EI Rev | CAAC*GAATTC*CAGAAGGTGACCATCACTATAGC |
| Mt SMG7.1 Ter BI For | CAAC*GGATCC*TGTCGGTTTAAGAATATTGACGGT |
| Mt SMG7.1 Ter EI Rev | CAAC*GAATTC*TTGCTACTACCTCCGTTCCT |
| Mt SMG7.2 Ter BI For | CAAC*GGATCC*TGTCAGTATGAGAGTATAGATGGT |
| Mt SMG7.2 Ter EI Rev | CAAC*GAATTC*TCACAGTTTTACTTAGTTATTAGGGT |
| Mt eRF1-1 Ter BI For | CAAC*GGATCC*CAATCAATGAAATGCTGCCTACCAC |
| Mt eRF1-1 Ter EI Rev | CAAC*GAATTC*CTAATTGAGGATCTCAACCACCA |
| **RT-qPCR Primers** |  |
| UPF1_qF1 | GGAAGCAGACAATGTTGTCGG |
| UPF1_qR1 | TGCCACAAATTGTGAGTCACAAC |
| UPF2_qF | TTGATCAGGAGCTCAAGGCTG |
| UPF2_qR | TTGCTTACGCCAGTATCCTCG |
| Mt_eRF1_qF | GAGCAGGAAGCCAAGCAGAG |
| Mt_eRF1_qR | CGCAAGATCCCACCTATCCC |
| Mt_SMG7_qF | TGAGCTCTCACTGCCTGAAG |
| Mt_SMG7_qR | TGCAAAACTGACGATGACTTCAC |
| **Primers used for editing** |  |
| MtUPF1_gF1 | cttGCTGACCCGTCTGGCTCCGC |
| MtUPF1_gF2 | cttGTGTTGGGGCTGGAGATCCC |
| MtUPF1_gR1 | aaacGCGGAGCCAGACGGGTCAG |
| MtUPF1_gR2 | aaacGGGATCTCCAGCCCCAACA |
| MtUPF1_g1_NGS_F | TCGTCGGCAGCGTCAGATGTGTATAAGAGACAGcACTTTCCTCGAATTCAACAC |
| MtUPF1_g1_NGS_R | GTCTCGTGGGCTCGGAGATGTGTATAAGAGACAGtGACCCGCTTCCACTTCCT |
| MtUPF1_g1_RFLP_R | ACAAAACCATTTTCGACACGA |
| MtUPF1_g2_NGS_F | TCGTCGGCAGCGTCAGATGTGTATAAGAGACAGcGAGCTACAGAGAGGGAGA |
| MtUPF1_g2_NGS_R | GTCTCGTGGGCTCGGAGATGTGTATAAGAGACAGcTTGAGTGGACTCGTCAATAAG |
| MtUPF1_g2_RFLP_R | CTAAAGTACATTACCTGCAACCT |
| MtUPF2_g1F | cttGAAGATGAATGTCGCAATGA |
| MtUPF2_g1R | aaacTCATTGCGACATTCATCTT |
| MtUPF2_g1_NGS_F | TCGTCGGCAGCGTCAGATGTGTATAAGAGACAGaTGGAAGATAAGGGTATGGATC |
| MtUPF2_g1_NGS_R | GTCTCGTGGGCTCGGAGATGTGTATAAGAGACAGtGTAATAAACACGTGAATCCCT |
| MtUPF2_g1_RFLP_F2 | ATGATGTTTACCACTCACTCG |
| MtUPF2_g2F | aaacTGGATTCCAGCATTAAACG |
| MtUPF2_g2R | cttGCGTTTAATGCTGGAATCCA |
| MtUPF2_g2_NGS_F | TCGTCGGCAGCGTCAGATGTGTATAAGAGACAGgATGTTCATTGCAGACACAGG |
| MtUPF2_g2_NGS_R | GTCTCGTGGGCTCGGAGATGTGTATAAGAGACAGtGCCTCACATATAGCTGCCAC |
| MtUPF2_g2_RFLP_F | GGCATTGGGATTTCATATCGTG |
| **Primers used for RNAi** |  |
| UPF1_GW_F | ggggacaagtttgtacaaaaaagcaggctGTTCGGGCAAAGCACAAG |
| UPF1_GW_R | ggggaccactttgtacaagaaagctgggtACTACACTTTCTGTCTTTGCAG |
| UPF2_GW_F | ggggacaagtttgtacaaaaaagcaggctGTGATACGAACGAACAACCC |
| UPF2_GW_R | ggggaccactttgtacaagaaagctgggtAGTGAAGCTGAGTAGTAGAATCG |
| GUS_GW_F | ggggacaagtttgtacaaaaaagcaggctTCACAGCCAAAAGCCAGAC |
| GUS_GW_R | ggggaccactttgtacaagaaagctgggtATCAGCACGTTATCGAATCC |

Letters in italics show the added restriction sites and normal letters show the additional nucleotides that facilitate cleavage of the PCR products or used in recombination reactions of the Gateway® Technology.

**Plant material, growth conditions and bacterial strains**

*M. truncatula* Jemalong A17 genotype was used for gene editing and RNA silencing (RNAi) experiments. *M. truncatula* seeds were germinated as previously described (Domonkos et al., 2017), and plants were grown in growth chambers at 24 °C under 16/8 h light/dark photoperiod. *Nicotiana benthamiana* plants, used for co-agroinfiltration assays, were grown in the greenhouse. Following agroinfiltration, plants were transferred to a growth chamber at 23 °C under 16/8 h light/dark photoperiod. For rhizobia inoculation, *Sinorhizobium medicae* strain WSM419 carrying the *LacZ* expressing pXLGD4 plasmid was used (Auriac & Timmers, 2007).

**Construction of the CRISPR/Cas9 and RNAi binary vectors**

Guide RNAs (gRNA) were designed using the online tool CRISPOR (http://crispor.tefor.net/) (Concordet & Haeussler, 2018) to target the *M. truncatula* *UPF1* and *UPF2* genes (all primers used in this study are listed above). To generate binary transformation vectors, annealed oligonucleotides with four nucleotide 5’ overhangs were assembled into the vector pKSE466_RR using Golden Gate cloning (Güngör et al., 2023).

RNAi constructs were generated by cloning PCR fragments of the coding regions of the *M. truncatula* *UPF1* (134 bp), *UPF2* (128 bp) and the bacterial *GUS* (fragment size 172 bp) genes into pDONR201 vector using Gateway cloning (Thermo Fisher Scientific). The fragments were then inserted into destination vector pKGW-RR_IR by LR clonase II-mediated recombination to create RNAi constructs expressing transcripts containing an inverted repeat separated by a 342 bp stuffer region.

**Transformation of *M. truncatula* plants**

Constructs were introduced into wild-type *M. truncatula* A17 roots by *Agrobacterium rhizogenes*-mediated hairy root transformation using a slightly modified version of a previously published protocol (Boisson-Dernier et al., 2001). Briefly, the root tips of the seedlings were removed and the cut surface was immersed into culture of *A. rhizogenes* strain Arqua-1, and then the seedlings were grown on agar plates containing Fahraeus medium. After one week, the seedlings were transferred to the same media supplemented with kanamycin (25 mg ml^-1^) for two weeks. Plants having red fluorescent transgenic roots were transferred to zeolite substrate (Geoproduct Kft., Mád, Hungary) complemented with Fahraeus solution for two additional weeks to recover, and then *S. medicae* inoculation was conducted. DNA or RNA samples were isolated from pooled transgenic roots before inoculation.

**Analyses of CRISPR/Cas9 edited roots**

Genomic DNA was isolated from individual transformed roots as previously described (Güngör et al., 2023). Editing efficiency was assessed by restriction fragment length polymorphisms (RFLP) analysis. PCR fragments spanning the target regions were amplified using RFLP-NGS primer pairs and the amplicons were digested with *Ssi*I (UPF1_g1), *Bsp*143I (UPF1_g2), and *Hinf*I (UPF2_g2), respectively. For UPF2_g1, PCR amplicons were mixed with equal amounts of fragments amplified from empty vector-transformed roots, heat-denatured, reannealed, and subsequently digested with mismatch recognizing T7 endonuclease (Shan et al., 2014). Digested fragments were separated in agarose gels and the approximate mutation frequencies were assessed based on the amount of partially or non-digested DNA that indicated disruption restriction enzyme recognition sites. For more detailed analysis, the UPF1_g2, UPF2_g1, and UPF2_g2 NGS-fragments were sequenced on Illumina Myseq platform (2x150 bp paired-end reads; min. coverage 10 000 reads/sample). The sequencing data were analyzed using the CRISPresso2 online tool (Clement et al., 2019).

**RNA isolations and RT-qPCR assays**

To assess RNA silencing efficiency and perform transcriptome analysis, total RNA was purified from transformed roots (showing red fluorescence) using TRI Reagent (Sigma). RNA samples were further purified using the Direct-zol RNA MiniPrep Kit (Zymo Research, Irvine, CA, USA) and treated with DNaseI on Zymo-Spin columns. For quantitative RT-PCR (RT-qPCR), cDNA was prepared from 1 µg total RNA. Three biological replicates were analyzed for RT-qPCR. The ubiquitin domain-containing gene MtrunA17_Chr3g0126781 was used as the reference gene. Data analysis was performed using the LightCycler 96 SW1.1 software.

**Rhizobia inoculation, monitoring early infection events and nodule number**

Infection events and nodule numbers were quantified on transformed roots. Plants with DsRed fluorescent roots were inoculated with the *lacZ* reporter-expressing *S. medicae* WSM419 strain. Rhizobia cultures grown in TA medium were pelleted and resuspended in sterile nitrogen-free Fahraeus media to the optical density of 0.1 (OD_600_). At 4 days post inoculation (dpi), transformed roots were harvested, fixed and stained as previously described (Kovacs et al., 2021). The stained roots were analyzed using a Zeiss Axioskop 40 light microscope with 10x-40x objectives and early infection events (root hair curling, infection thread formation) were quantified. Nodule numbers were counted after 4 weeks post-inoculation (wpi).

**Comparative transcriptome analyses**

To analyze the transcriptome changes associated with NMD-inactivation in roots, comparative poly(A) enriched RNA-sequencing (RNA-seq) was conducted using total RNA isolated from empty vector (EV) control and *upf1* and *upf2* RNAi test roots, immediately prior to *S. medicae* inoculation. Three biological replicates were analyzed per condition, each consisting of pooled transgenic roots selected based on red fluorescence. RNA-seq and part of the subsequent bioinformatics analysis were performed out the Novogene Company. Bioinformatical analysis related to NMD features and their enrichment were performed in-house.

Library preparation and sequencing: RNA integrity was assessed using the Bioanalyzer 2100 system (Agilent Technologies). mRNA was enriched from total RNA using poly(T) oligo-attached magnetic beads. Following fragmentation, first-strand cDNA was synthesized using random hexamer primers. The resulting cDNA underwent end repair, A-tailing, adapter ligation, size selection, amplification, and purification to generate the final libraries. Library quality was validated using Qubit and real-time PCR for quantification, and fragment size distribution was assessed using a Bioanalyzer. Libraries were sequenced on an Illumina platform using a "Sequencing by Synthesis" approach, generating more than 40 million reads per sample.

Bioinformatics analysis: Raw sequencing data in fastq format were processed using fastp software to generate clean data by removing reads containing adapters, poly-N sequences, and low-quality reads. The cleaned reads had a quality score of Q30>99.9%. The clean paired-end reads were aligned to the *M. truncatula* reference genome (ensembl_60_medicago_truncatula_mtruna17r5_0_anr_toplevel) using Hisat2 v2.0.5, which was selected for its capability to generate a splice junction database based on gene model annotations. The mapping efficiency of reads exceeded 93% for all samples, and more than 98% of mapped reads were localized to exonic regions. Mapped reads were subsequently assembled using StringTie (v1.3.3b) via a reference-based approach to predict novel transcripts. Gene expression levels were quantified using feature Counts v1.5.0-p3 to count reads mapped to each gene. To normalize for sequencing depth and gene length, the FPKM (Fragments Per Kilobase of transcript per Million mapped reads) was calculated for each gene. Differential expression analysis was performed using DESeq2 (1.20.0). P-values were adjusted using the Benjamini and Hochberg method to control the false discovery rate. Significant differential expression (relative to the EV control) was defined by a corrected P-value (padj≤0.05) and an absolute log2 (fold change) ≥1. For all *M. truncatula* genes, 3'UTR characteristics (length and exon structure) and 5'UTR uORF features were collected by parsing the reference genome sequence and annotation file with in-house software. During uORF identification, a start codon was considered to be in Kozak context if A or G was at -3 and G at +4 positions. Statistical calculations (Wilcoxon test, odds ratio, confidence interval, Fisher exact test) used in various NMD feature overrepresentation investigations as well as principal component analysis (PCA) were also performed by self-developed R and Python scripts. Gene Ontology (GO) and KEGG pathway enrichment analyses were conducted on the differentially expressed genes using the clusterProfiler R package. GO terms and KEGG pathways with p<0.05 and with padj<0.05(corrected P-value) were considered enriched and strongly enriched, respectively.

We have also analyzed the expression of NMD genes and the identified DEGs during the early phase of RNS. For this purpose a publicly available dataset from the SRA database (NCBI Bioproject ID: PRJNA524899) was downloaded (Poehlman et al., 2019). Raw sequences were processed with fastp (v0.20.1) to remove adapters, homopolymer sequences, and low quality bases. The processed sequences were pseudoaligned to the *M. truncatula* reference transcriptome (MtrunA17r5.0-ANR-EGN-r1.6.mrna.fasta) and quantified with kallisto (v0.46.1) and sleuth (v0.30.2). During the analysis, genes with no expression were filtered out. To identify genes where the interaction between Time and Treatment significantly affected expression, we performed a Likelihood Ratio Test (LRT) using the tool sleuth. The full model (Time + Treatment + Time:Treatment) was compared to the reduced model (Time + Treatment). False discovery rate (FDR) was controlled the using the Benjamini-Hochberg procedure. Genes with adjusted p-value (padj) < 0.1 (10% FDR) were considered significantly differentially expressed. From this set, NMD factors as well as genes up- and downregulated in *upf1* were selected for further analysis.

**Cloning of the terminator regions and the UPF1 dominant-negative assay**

To assess the NMD sensitivity of the 3’UTRs of *M. truncatula* NMD factors (MtUPF1, MtUPF2, MtUPF3, MtSMG7.1 and MtSMG7.2), the putative terminator regions (sequence from the stop codon to 200 nt downstream of the annotated polyadenylation/cleavage site) were PCR amplified and digested by BamHI and EcoRI. The terminator region of the GFP600 vector was replaced with the corresponding *M. truncatula* NMD terminators (G-MtU1T, G-MtU2T, G-MtU3T, G-MtS7.1T, G-MtS7.2T). UPF1 dominant-negative (U1DN) co-agroinfiltration assays were conducted as described (Kerényi et al., 2008; Kertész et al., 2006). Briefly, constructs were transformed into an *Agrobacterium tumefaciens*, and then *N. benthamiana* leaves were agroinfiltrated with a mixture of liquid bacterium cultures diluted to OD 0.3 (except the Myc-P14, which was diluted to 0.2). The test construct was co-agroinfiltrated with the Myc-P14 in the presence or absence of the U1DN. The Myc-P14 has a dual role in the assay, acting both as an RNA silencing suppressor that prevents the induction of agroinfiltration-induced RNA silencing and as an internal control (Nyikó et al., 2017). U1DN efficiently inhibits NMD in *N. benthamiana*. The U1DN, the Myc-P14 and NMD sensitive GFP600 control constructs were described previously (Kertész et al., 2006; Nyikó et al., 2017).

**References**

Auriac, M. C., & Timmers, A. C. J. (2007). Nodulation studies in the model legume Medicago truncatula: Advantages of using the constitutive EF1α promoter and limitations in detecting fluorescent reporter proteins in nodule tissues. *Molecular Plant-Microbe Interactions*, *20*(9), 1040–1047. https://doi.org/10.1094/MPMI-20-9-1040,

Andrio, E., Marino, D., Marmeys, A., de Segonzac, M. D., Damiani, I., Genre, A., Huguet, S., Frendo, P., Puppo, A., & Pauly, N. (2013). Hydrogen peroxide-regulated genes in the Medicago truncatula-Sinorhizobium meliloti symbiosis. *The New Phytologist*, *198*(1), 179–189. https://doi.org/10.1111/NPH.12120

Asai, T., Tena, G., Plotnikova, J., Willmann, M. R., Chiu, W. L., Gomez-Gomez, L., Boller, T., Ausubel, F. M., & Sheen, J. (2002). Map kinase signalling cascade in Arabidopsis innate immunity. *Nature*, *415*(6875), 977–983. https://doi.org/10.1038/415977a

Boisson-Dernier, A., Chabaud, M., Garcia, F., Bécard, G., Rosenberg, C., & Barker, D. G. (2001). Agrobacterium rhizogenes-transformed roots of Medicago truncatula for the study of nitrogen-fixing and endomycorrhizal symbiotic associations. *Molecular Plant-Microbe Interactions*, *14*(6), 695–700. https://doi.org/10.1094/MPMI.2001.14.6.695,

Clement, K., Rees, H., Canver, M. C., Gehrke, J. M., Farouni, R., Hsu, J. Y., Cole, M. A., Liu, D. R., Joung, J. K., Bauer, D. E., & Pinello, L. (2019). CRISPResso2 provides accurate and rapid genome editing sequence analysis. *Nature Biotechnology*, *37*(3), 224–226. https://doi.org/10.1038/S41587-019-0032-3,

Degtiar, E., Fridman, A., Gottlieb, D., Vexler, K., Berezin, I., Farhi, R., Golani, L., & Shaul, O. (2015). The feedback control of UPF3 is crucial for RNA surveillance in plants. *Nucleic Acids Research*, *43*(8), 4219–4235. https://doi.org/10.1093/NAR/GKV237

Domonkos, Á., Kovács, S., Gombár, A., Kiss, E., Horváth, B., Kováts, G. Z., Farkas, A., Tóth, M. T., Ayaydin, F., Bóka, K., Fodor, L., Ratet, P., Kereszt, A., Endre, G., & Kaló, P. (2017). NAD1 Controls Defense-Like Responses in Medicago truncatula Symbiotic Nitrogen Fixing Nodules Following Rhizobial Colonization in a BacA-Independent Manner. *Genes*, *8*(12). https://doi.org/10.3390/genes8120387

Feng, Y., Wu, P., Liu, C., Peng, L., Wang, T., Wang, C., Tan, Q., Li, B., Ou, Y., Zhu, H., Yuan, S., Huang, R., Stacey, G., Zhang, Z., & Cao, Y. (2021). Suppression of LjBAK1-mediated immunity by SymRK promotes rhizobial infection in Lotus japonicus. *Molecular Plant*, *14*(11), 1935–1950. https://doi.org/10.1016/J.MOLP.2021.07.016

Gloggnitzer, J., Akimcheva, S., Srinivasan, A., Kusenda, B., Riehs, N., Stampfl, H., Bautor, J., Dekrout, B., Jonak, C., Jiménez-Gómez, J. M., Parker, J. E., & Riha, K. (2014). Nonsense-mediated mRNA decay modulates immune receptor levels to regulate plant antibacterial defense. *Cell Host and Microbe*, *16*(3), 376–390. https://doi.org/10.1016/j.chom.2014.08.010

Güngör, B., Biró, J. B., Domonkos, Á., Horváth, B., & Kaló, P. (2023). Targeted mutagenesis of Medicago truncatula Nodule-specific Cysteine-Rich (NCR) genes using the Agrobacterium rhizogenes-mediated CRISPR/Cas9 system. *Scientific Reports*, *13*(1). https://doi.org/10.1038/s41598-023-47608-5

Kerényi, Z., Mérai, Z., Hiripi, L., Benkovics, A., Gyula, P., Lacomme, C., Barta, E., Nagy, F., & Silhavy, D. (2008). Inter-kingdom conservation of mechanism of nonsense-mediated mRNA decay. *EMBO Journal*, *27*(11), 1585–1595. https://doi.org/10.1038/emboj.2008.88

Kertész, S., Kerényi, Z., Mérai, Z., Bartos, I., Pálfy, T., Barta, E., & Silhavy, D. (2006). Both introns and long 3′-UTRs operate as cis-acting elements to trigger nonsense-mediated decay in plants. *Nucleic Acids Research*, *34*(21), 6147–6157. https://doi.org/10.1093/nar/gkl737

Kovacs, S., Fodor, L., Domonkos, A., Ayaydin, F., Laczi, K., Rákhely, G., & Kalo, P. (2021). Amino Acid Polymorphisms in the VHIID Conserved Motif of Nodulation Signaling Pathways 2 Distinctly Modulate Symbiotic Signaling and Nodule Morphogenesis in Medicago truncatula. *Frontiers in Plant Science*, *12*, 709857. https://doi.org/10.3389/fpls.2021.709857

Luo, Z., Wang, J., Li, F., Lu, Y., Fang, Z., Fu, M., Mysore, K. S., Wen, J., Gong, J., Murray, J. D., & Xie, F. (2023). The small peptide CEP1 and the NIN-like protein NLP1 regulate NRT2.1 to mediate root nodule formation across nitrate concentrations. *The Plant Cell*, *35*(2), 776–794. https://doi.org/10.1093/PLCELL/KOAC340

Nyikó, T., Auber, A., Szabadkai, L., Benkovics, A., Auth, M., Mérai, Z., Kerényi, Z., Dinnyés, A., Nagy, F., & Silhavy, D. (2017). Expression of the eRF1 translation termination factor is controlled by an autoregulatory circuit involving readthrough and nonsense-mediated decay in plants. *Nucleic Acids Research*, *45*(7), 4174–4188. https://doi.org/10.1093/nar/gkw1303

Nyikó, T., Kerényi, F., Szabadkai, L., Benkovics, A. H., Major, P., Sonkoly, B., Mérai, Z., Barta, E., Niemiec, E., Kufel, J., & Silhavy, D. (2013). Plant nonsense-mediated mRNA decay is controlled by different autoregulatory circuits and can be induced by an EJC-like complex. *Nucleic Acids Research*, *41*(13), 6715–6728. https://doi.org/10.1093/nar/gkt366

Rawat, A. A., Hartmann, M., Harzen, A., Lugan, R., Stolze, S. C., Forzani, C., Abts, L., Reißenweber, S., Rayapuram, N., Nakagami, H., Zeier, J., & Hirt, H. (2023). OXIDATIVE SIGNAL-INDUCIBLE1 induces immunity by coordinating N-hydroxypipecolic acid, salicylic acid, and camalexin synthesis. *The New Phytologist*, *237*(4), 1285–1301. https://doi.org/10.1111/NPH.18592

Rayson, S., Arciga-Reyes, L., Wootton, L., de Torres Zabala, M., Truman, W., Graham, N., Grant, M., & Davies, B. (2012). A role for nonsense-mediated mrna decay in plants: Pathogen responses are induced in arabidopsis thaliana nmd mutants. *PLoS ONE*, *7*(2). https://doi.org/10.1371/journal.pone.0031917

Raza, A., Charagh, S., Zahid, Z., Mubarik, M. S., Javed, R., Siddiqui, M. H., & Hasanuzzaman, M. (2021). Jasmonic acid: a key frontier in conferring abiotic stress tolerance in plants. *Plant Cell Reports*, *40*(8), 1513–1541. https://doi.org/10.1007/s00299-020-02614-z

Ryu, H., Laffont, C., Frugier, F., & Hwang, I. (2017). Map kinase-mediated negative regulation of symbiotic nodule formation in Medicago truncatula. *Molecules and Cells*, *40*(1), 17–23. https://doi.org/10.14348/MOLCELLS.2017.2211

Poehlman, W. L., Schnabel, E. L., Chavan, S. A., Frugoli, J. A., & Feltus, F. A. (2019). Identifying Temporally Regulated Root Nodulation Biomarkers Using Time Series Gene Co-Expression Network Analysis. *Frontiers in Plant Science*, *10*, 1409. https://doi.org/10.3389/fpls.2019.01409

Saifi, F., Biró, J. B., Horváth, B., Vizler, C., Laczi, K., Rákhely, G., Kovács, S., Kang, M., Li, D., Chen, Y., Chen, R., Domonkos, Á., & Kaló, P. (2024). Two members of a Nodule‐specific Cysteine‐Rich (NCR) peptide gene cluster are required for differentiation of rhizobia in *Medicago truncatula* nodules. *The Plant Journal*, tpj.16871. https://doi.org/10.1111/tpj.16871

Shan, Q., Wang, Y., Li, J., & Gao, C. (2014). Genome editing in rice and wheat using the CRISPR/Cas system. *Nature Protocols*, *9*(10), 2395–2410. https://doi.org/10.1038/NPROT.2014.157,

Andrio, E., Marino, D., Marmeys, A., de Segonzac, M. D., Damiani, I., Genre, A., Huguet, S., Frendo, P., Puppo, A., & Pauly, N. (2013). Hydrogen peroxide-regulated genes in the Medicago truncatula-Sinorhizobium meliloti symbiosis. *The New Phytologist*, *198*(1), 179–189. https://doi.org/10.1111/NPH.12120

Asai, T., Tena, G., Plotnikova, J., Willmann, M. R., Chiu, W. L., Gomez-Gomez, L., Boller, T., Ausubel, F. M., & Sheen, J. (2002). Map kinase signalling cascade in Arabidopsis innate immunity. *Nature*, *415*(6875), 977–983. https://doi.org/10.1038/415977a

Calle García, J., Guadagno, A., Paytuvi-Gallart, A., Saera-Vila, A., Amoroso, C. G., D’esposito, D., Andolfo, G., Aiese Cigliano, R., Sanseverino, W., Ercolano, M. R., & Sanseverino, W. (2022). PRGdb 4.0: an updated database dedicated to genes involved in plant disease resistance process. *Nucleic Acids Research*, *50*(D1), D1483–D1490. https://doi.org/10.1093/NAR/GKAB1087

Degtiar, E., Fridman, A., Gottlieb, D., Vexler, K., Berezin, I., Farhi, R., Golani, L., & Shaul, O. (2015). The feedback control of UPF3 is crucial for RNA surveillance in plants. *Nucleic Acids Research*, *43*(8), 4219–4235. https://doi.org/10.1093/NAR/GKV237

Feng, Y., Wu, P., Liu, C., Peng, L., Wang, T., Wang, C., Tan, Q., Li, B., Ou, Y., Zhu, H., Yuan, S., Huang, R., Stacey, G., Zhang, Z., & Cao, Y. (2021). Suppression of LjBAK1-mediated immunity by SymRK promotes rhizobial infection in Lotus japonicus. *Molecular Plant*, *14*(11), 1935–1950. https://doi.org/10.1016/J.MOLP.2021.07.016

Gloggnitzer, J., Akimcheva, S., Srinivasan, A., Kusenda, B., Riehs, N., Stampfl, H., Bautor, J., Dekrout, B., Jonak, C., Jiménez-Gómez, J. M., Parker, J. E., & Riha, K. (2014). Nonsense-mediated mRNA decay modulates immune receptor levels to regulate plant antibacterial defense. *Cell Host and Microbe*, *16*(3), 376–390. https://doi.org/10.1016/j.chom.2014.08.010

Luo, Z., Wang, J., Li, F., Lu, Y., Fang, Z., Fu, M., Mysore, K. S., Wen, J., Gong, J., Murray, J. D., & Xie, F. (2023). The small peptide CEP1 and the NIN-like protein NLP1 regulate NRT2.1 to mediate root nodule formation across nitrate concentrations. *The Plant Cell*, *35*(2), 776–794. https://doi.org/10.1093/PLCELL/KOAC340

Nyikó, T., Auber, A., Szabadkai, L., Benkovics, A., Auth, M., Mérai, Z., Kerényi, Z., Dinnyés, A., Nagy, F., & Silhavy, D. (2017). Expression of the eRF1 translation termination factor is controlled by an autoregulatory circuit involving readthrough and nonsense-mediated decay in plants. *Nucleic Acids Research*, *45*(7), 4174–4188. https://doi.org/10.1093/nar/gkw1303

Nyikó, T., Kerényi, F., Szabadkai, L., Benkovics, A. H., Major, P., Sonkoly, B., Mérai, Z., Barta, E., Niemiec, E., Kufel, J., & Silhavy, D. (2013). Plant nonsense-mediated mRNA decay is controlled by different autoregulatory circuits and can be induced by an EJC-like complex. *Nucleic Acids Research*, *41*(13), 6715–6728. https://doi.org/10.1093/nar/gkt366

Rawat, A. A., Hartmann, M., Harzen, A., Lugan, R., Stolze, S. C., Forzani, C., Abts, L., Reißenweber, S., Rayapuram, N., Nakagami, H., Zeier, J., & Hirt, H. (2023). OXIDATIVE SIGNAL-INDUCIBLE1 induces immunity by coordinating N-hydroxypipecolic acid, salicylic acid, and camalexin synthesis. *The New Phytologist*, *237*(4), 1285–1301. https://doi.org/10.1111/NPH.18592

Rayson, S., Arciga-Reyes, L., Wootton, L., de Torres Zabala, M., Truman, W., Graham, N., Grant, M., & Davies, B. (2012). A role for nonsense-mediated mrna decay in plants: Pathogen responses are induced in arabidopsis thaliana nmd mutants. *PLoS ONE*, *7*(2). https://doi.org/10.1371/journal.pone.0031917

Raza, A., Charagh, S., Zahid, Z., Mubarik, M. S., Javed, R., Siddiqui, M. H., & Hasanuzzaman, M. (2021). Jasmonic acid: a key frontier in conferring abiotic stress tolerance in plants. *Plant Cell Reports*, *40*(8), 1513–1541. https://doi.org/10.1007/s00299-020-02614-z

Ryu, H., Laffont, C., Frugier, F., & Hwang, I. (2017). Map kinase-mediated negative regulation of symbiotic nodule formation in Medicago truncatula. *Molecules and Cells*, *40*(1), 17–23. https://doi.org/10.14348/MOLCELLS.2017.2211

Sugimoto, K., Allmann, S., & Kolomiets, M. V. (2022). Editorial: Oxylipins: The Front Line of Plant Interactions. *Frontiers in Plant Science*, *13*, 878765. https://doi.org/10.3389/FPLS.2022.878765

Sun, J., Cardoza, V., Mitchell, D. M., Bright, L., Oldroyd, G., & Harris, J. M. (2006). Crosstalk between jasmonic acid, ethylene and Nod factor signaling allows integration of diverse inputs for regulation of nodulation. *The Plant Journal : For Cell and Molecular Biology*, *46*(6), 961–970. https://doi.org/10.1111/J.1365-313X.2006.02751.X

Tsikou, D., Ramirez, E. E., Psarrakou, I. S., Wong, J. E., Jensen, D. B., Isono, E., Radutoiu, S., & Papadopoulou, K. K. (2018). A Lotus japonicus E3 ligase interacts with the Nod Factor Receptor 5 and positively regulates nodulation. *BMC Plant Biology*, *18*(1). https://doi.org/10.1186/S12870-018-1425-Z

Varma Penmetsa, R., Uribe, P., Anderson, J., Lichtenzveig, J., Gish, J. C., Nam, Y. W., Engstrom, E., Xu, K., Sckisel, G., Pereira, M., Baek, J. M., Lopez-Meyer, M., Long, S. R., Harrison, M. J., Singh, K. B., Kiss, G. B., & Cook, D. R. (2008). The Medicago truncatula ortholog of Arabidopsis EIN2, sickle, is a negative regulator of symbiotic and pathogenic microbial associations. *The Plant Journal : For Cell and Molecular Biology*, *55*(4), 580–595. https://doi.org/10.1111/J.1365-313X.2008.03531.X

Vexler, K., Cymerman, M. A., Berezin, I., Fridman, A., Golani, L., Lasnoy, M., Saul, H., & Shaul, O. (2016). The Arabidopsis NMD factor UPF3 is feedback-regulated at multiple levels and plays a role in plant response to salt stress. *Frontiers in Plant Science*, *7*(September2016). https://doi.org/10.3389/FPLS.2016.01376

Wang, Z., Wei, X., Wang, Y., Sun, M., Zhao, P., Wang, Q., Yang, B., Li, J., & Jiang, Y. Q. (2023). *WRKY29 transcription factor regulates ethylene biosynthesis and response in arabidopsis*. *194*, 134–145. https://pubmed.ncbi.nlm.nih.gov/36403487/

Wu, W., Zhuang, Y., Chen, D., Ruan, Y., Li, F., Jackson, K., Liu, C. W., East, A., Wen, J., Tatsis, E., Poole, P. S., Xu, P., & Murray, J. D. (2024). Methylated chalcones are required for rhizobial nod gene induction in the Medicago truncatula rhizosphere. *The New Phytologist*, *242*(5), 2195–2206. https://doi.org/10.1111/NPH.19701

Wu, Y., Ma, Y., Wang, W., Zhang, S., & Wu, W. (2025). Mastering the balance: BAK1’s dual roles in steering plant growth and immunity. *Horticulture Research*, *12*(11). https://doi.org/10.1093/HR/UHAF206

Yang, Q., & Wang, G. (2024). Isoflavonoid metabolism in leguminous plants: an update and perspectives. *Frontiers in Plant Science*, *15*. https://doi.org/10.3389/FPLS.2024.1368870

Zhai, J., Jeong, D. H., de Paoli, E., Park, S., Rosen, B. D., Li, Y., González, A. J., Yan, Z., Kitto, S. L., Grusak, M. A., Jackson, S. A., Stacey, G., Cook, D. R., Green, P. J., Sherrier, D. J., & Meyers, B. C. (2011). MicroRNAs as master regulators of the plant NB-LRR defense gene family via the production of phased, trans-acting siRNAs. *Genes and Development*, *25*(23), 2540–2553. https://doi.org/10.1101/gad.177527.111
